# Effect of Plant Proteases on Oxidative Stability, Physicochemical and Sensory Properties of Horse Meat from Different Age Groups During Marination

**DOI:** 10.3390/molecules31183236

**Published:** 2026-09-13

**Authors:** Renata Stanisławczyk, Mariusz Rudy, Marian Gil

**Affiliations:** Department of Agricultural Processing and Commodity Science, Institute of Food and Nutrition Technology, Faculty of Technology and Life Sciences, University of Rzeszow, Zelwerowicza 4, 35-601 Rzeszów, Poland; mrudy@ur.edu.pl (M.R.); mgil@ur.edu.pl (M.G.)

**Keywords:** papain, bromelain, TBARS, redox potential, physicochemical properties, textural profile, lipid oxidation, oxidative stability, sensory properties

## Abstract

The aim of this study was to evaluate the effect of plant proteases on the oxidative stability as well as the physicochemical and sensory properties of horse meat derived from different age groups during marination. The experimental material consisted of samples of the *longissimus thoracis muscle* obtained from 24 horse half-carcasses, with 12 half-carcass representing horses aged 2–5 years and 12 half-carcass representing horses aged 8–12 years. Meat samples were treated with papain (P), bromelain (B), and actinidin (A) solutions at a concentration of 25 mg/100 g, as well as with a 0.5% culinary tenderizer solution (MT). The applied treatments significantly improved meat quality by reducing shear force, hardness, stiffness, and chewiness, and by affecting color parameters, including increased lightness (L*) and redness (a*) in both age groups (*p* < 0.05). At the same time, decreases in TBARS values, oxidation–reduction potential (ORP), and forced drip loss were observed, alongside an increase in cooking loss with extended marination time. Higher TBARS values were recorded in meat obtained from older animals, indicating a greater susceptibility to lipid oxidation. Prolonged marination, particularly up to 48 h, contributed to a reduction in lipid peroxidation processes.

## 1. Introduction

Marinating has been a key meat processing technique for centuries. Traditionally, the process involves immersing meat in solutions containing salt, sugar, acids (e.g., acetic, tartaric, or lactic), spices, and other supporting ingredients such as herbs [1]. Marinating improves the flavor, aroma, and texture of meat, and extends its shelf life by reducing lipid oxidation and the growth of undesirable microorganisms [2,3,4,5].

One of the most innovative trends in meat marinating is the use of plant enzymes (proteases, also called proteinases or proteolytic enzymes). The main enzymes used for this purpose are: papain found in the latex of the papaya plant (*Carica papaya*), bromelain contained in pineapple (*Ananas comosus*), actinidin present in kiwi fruit (*Actinidia deliciosa*) and mini kiwi (*Actinidia arguta*), ficin derived from the latex of fig trees (*Ficus carica* L.), zingibain found in the rhizomes of ginger (*Zingiber officinale*), Bacillus Protease and Aspartic Protease. These proteolytic enzymes are recognized as safe for use in food applications, having been granted GRAS status by the USDA Food Safety and Inspection Service [6,7,8]. Plant proteases are enzymes that catalyze the hydrolysis of peptide bonds in proteins [1,9]. In meat tenderization, their activity is mainly related to protein denaturation, destabilization of the actomyosin complex, and controlled proteolysis. As a consequence, long myofibrillar protein chains are fragmented, which may partially reduce the rigidity and toughness of the muscle structure. Moreover, treatment with plant-derived enzymes decreases the content of high-molecular-weight proteins while substantially increasing the amount of low-molecular-weight protein compounds [10]. Plant proteases, through their ability to break down meat proteins, increase the meat’s tenderness and shorten the cooking time [8]. These enzymes primarily degrade connective tissue proteins—collagen and elastin—and only slightly affect muscle fibers [11]. This enzymatic action is particularly useful when processing tougher meats, such as beef or game, which, after proper marinating, become much juicier and easier to prepare [12,13]. Plant-derived proteases, such as papain, bromelain, and actinidin, may contribute to oxidative stability of meat through proteolytic activity and through bioactive compounds associated with their fruit sources. According to previous studies, plant-derived materials have been associated with antioxidant effects. However, the mechanisms responsible for these effects are beyond the scope of the present study [14,15]. Additionally, these fruits enrich marinades with valuable vitamins, including vitamin C, which has antioxidant properties and supports the body’s immune system. In addition to the enzyme actinidin, mini kiwi also contains B vitamins, minerals (such as calcium, potassium, and zinc), and polyphenols, which have been reported to exhibit antioxidant properties [12,13]. Marinated meat, thanks to the enzymatic action of fruit extracts, does not require such high temperatures or long processing times, which translates into reduced nutrient losses and a reduced risk of the formation of harmful substances such as polycyclic aromatic hydrocarbons and heterocyclic aromatic amines. Furthermore, incorporating fruit extracts into marinades helps preserve more vitamins and minerals that might otherwise be destroyed during the prolonged heat treatment [8,12,13].

Papain is one of the most commonly used exogenous plant proteases for meat tenderization, which results from its ability to hydrolyze both myofibrillar proteins and connective tissue structures. Its high thermal stability makes it relatively resistant to inactivation, enabling continued modifications in meat texture even after cooking [14]. Previous studies evaluated the application of papain for beef tenderization at concentrations of 0.003, 0.005, 0.007, and 0.01 mg/100 g of meat, subjecting the meat to enzymatic action for 24–48 h at 4 °C prior to thermal processing (83 °C for 10 min). Increasing the application time and increasing the enzyme concentration resulted in increased tenderness and juiciness of the beef [16]. The profile of enzymatically treated muscle proteins indicates that papain is an enzyme that degrades myosin and actin at a similar rate, whereas bromelain degrades mainly myosin and troponin T without affecting actin, resulting in the formation of smaller protein fragments [17]. Therefore, the enzymatic activity of bromelain is somewhat more limited than that of papain [18]. This fact has been confirmed by studies [19] in which after the application of various concentrations of papain and bromelain (10, 15, and 20 mg of enzyme/100 g of meat to beef for 24 and 48 h at 4 °C), the meat tenderization process was more noticeable after the use of papain. Actinidin, on the other hand, is an enzyme that hydrolyzes myofibrillar proteins, thus leading to the formation of new peptides and the activation of m-calpain during postmortem aging [20]. According to Aminlari et al. [21], actinidin, compared to other enzymes such as papain, does not hydrolyze as many myofibrillar proteins and can therefore be used when a moderate level of muscle tissue softening is required. Zhang et al. [22] evaluated the tenderizing potential of crude and purified actinidin preparations in pork and rabbit *Longissimus dorsi* muscles using enzyme concentrations of 0.25 and 0.5 mg/100 g. The meat samples were incubated with actinidin for 3 h at 20 °C and then cooked at 75 °C for 30 min. Compared with papain-treated samples, those exposed to actinidin showed higher shear force values, indicating a less pronounced tenderizing effect, especially in pork meat. 

Horse age can influence both the technological and sensory qualities of horse meat. As horses age, the water content in the meat decreases, while the fat, macronutrient, and micronutrient contents increase [23]. The increase in collagen content with age causes the meat to become tougher and its nutritional value to decline. Consequently, horse meat from older animals is more fibrous and tough, even after heat treatment. Meat quality is adversely affected by the fact that the slaughter yield decreases with age and the fat content increases. Longevity also affects the color of horse meat. Meat darkens with age, and fat takes on a yellowish, sometimes even orange, hue [24]. 

This raw meat requires a longer maturation period under refrigerated conditions, during which the horse meat darkens due to the oxidation of myoglobin to metmyoglobin (Mb^3+^), a brown pigment. As a result, the meat loses brightness and becomes dull and brownish-gray.

The susceptibility of horse meat to oxidative deterioration is largely associated with its fatty acid profile, which contains relatively high levels of essential polyunsaturated fatty acids (PUFAs), including α-linolenic acid. The high PUFA content makes horse meat more vulnerable to free radical attack than beef or pork [25,26]. An additional factor increasing its oxidative susceptibility is the high concentration of myoglobin and associated heme iron, which can catalyze lipid and protein oxidation reactions. A key factor determining the oxidative stability of horse meat is the age of the animal at slaughter [27]. As horses age, significant biochemical changes occur within muscle tissue. The concentration of myoglobin and associated heme iron increases progressively with animal maturity, reaching the highest levels in older horses. Since iron is one of the major catalysts of the Fenton reaction, leading to the formation of highly reactive hydroxyl radicals, meat from older horses exhibits a greater propensity for autoxidation compared with that of young foals [27,28,29].

Moreover, intramuscular fat content tends to increase with age; therefore, the coexistence of higher levels of unsaturated lipids and elevated concentrations of pro-oxidative heme iron creates an environment that favors the intensification of lipid peroxidation and the generation of secondary oxidation products [25,26,27].

Reactive oxygen species (ROS) and free radicals initiate a series of detrimental changes in muscle tissue that are responsible for reducing the nutritional value and overall quality of meat. Among these processes, lipid peroxidation plays a central role as a free radical chain reaction consisting of three stages: initiation, propagation, and termination. During the initiation phase, external factors such as temperature and light, together with the heme iron naturally present in horse meat, promote the abstraction of a hydrogen atom from a polyunsaturated fatty acid (PUFA), resulting in the formation of an unstable and highly reactive lipid radical. During the propagation phase, the lipid radical reacts with molecular oxygen to generate a peroxyl radical, which subsequently attacks neighboring intact lipid molecules. As a consequence, primary oxidation products, namely lipid hydroperoxides, together with additional free radicals, are formed, giving the reaction its self-propagating chain characteristic. In the final stage (termination), the reaction chain is quenched when the concentration of free radicals becomes sufficiently high to promote their mutual interaction and recombination into more stable, non-radical compounds, such as dimers [30,31,32].

Lipid hydroperoxides undergo further degradation to secondary oxidation products, among which malondialdehyde (MDA), a cytotoxic aldehyde, is one of the most prominent. These compounds are responsible for lipid rancidity and the development of off-flavors, discoloration resulting from the oxidation of myoglobin to brown metmyoglobin, and deterioration of meat texture manifesting as increased dryness and toughness due to the carbonylation of structural proteins [30,31,33,34]. 

The incorporation of exogenous compounds with antioxidant potential, such as tocopherols, botanical polyphenols, and preparations containing plant proteolytic enzymes (e.g., papain, bromelain, or actinidin), may contribute to preserving the quality of horse meat, particularly that derived from older animals [14,27,35,36,37]. Antioxidants act primarily during the propagation stage by donating a hydrogen atom or an electron to lipid radicals, thereby interrupting the autoxidative chain reaction [26,37]. Previous studies suggest that proteolytic treatment may influence oxidative stability; however, the underlying mechanisms were not investigated in the present work [14,34].

After reviewing the available literature on the effects of plant-derived enzymes on the quality of various types of meat, it can be concluded that studies focusing on horse meat remain very limited [37,38,39]. Therefore, the aim of the present study was to investigate the effects of selected plant proteases on horse meat quality, with particular emphasis on oxidative stability, texture characteristics, and physicochemical and sensory properties.

The present study contributes to the understanding of the effects of plant proteases, marination time, and animal age on horse meat quality. Available information regarding the simultaneous assessment of oxidative changes using TBARSs and ORP in enzymatically marinated horse meat appears to be limited. Therefore, the present study expands the current knowledge by examining both indicators under the same experimental conditions. Another innovative feature of the study is the inclusion of two age groups of horses, allowing for the evaluation of how animal age—one of the key factors determining horse meat quality—influences the effectiveness of individual proteolytic enzymes.

An additional scientific contribution of this work is the application of two marination periods (24 and 48 h), which enabled the assessment of the dynamics of quality changes occurring in the meat as a function of enzyme exposure time. This approach made it possible not only to compare the effectiveness of different proteolytic preparations (papain, bromelain, and actinidin), but also to investigate the interactions among enzyme type, marination time, and animal age. The combined evaluation of enzyme type, marination time, and animal age provides additional information regarding factors affecting horse meat quality and highlights the practical significance of the present study.

Furthermore, while horse age is known to significantly affect meat quality and technological properties, studies combining age-related differences with enzymatic marination strategies remain scarce. Therefore, the simultaneous evaluation of two horse age groups, three plant-derived proteases, and two marination times provides a multidimensional understanding of the tenderization process and its impact on horse meat quality, offering valuable knowledge for both the scientific community and the meat industry.

## 2. Results and Discussion

### 2.1. Chemical Composition

As shown in Table 1, the chemical composition of the horse meat was significantly influenced by animal age, particularly in relation to fat and water contents (*p* < 0.05). Across all marinating treatments and marination times, older animals generally exhibited higher fat levels and lower moisture content. Similar age-dependent changes in horse meat composition have been described in previous studies, which reported increases in protein and fat contents accompanied by a reduction in water content with advancing age [40,41,42]. However, there is still a lack of comprehensive data on how plant-derived proteases affect the proximate composition of meat. Orynbekov et al. [37] demonstrated that the content of basic components in horse meat did not change significantly (*p* > 0.05) depending on the use and concentration of the bromelain enzyme solution. The cited authors used bromelain in powder form and in an aqueous solution, with concentrations ranging from 0.5% to 2.5%.

### 2.2. Physicochemical Properties

Table 2 presents data on the physicochemical properties of the horse meat. These data indicate that the interaction between animal age, the type of substance used, and marinating time had a statistically significant effect on the pH variation of the horse meat. As the age of the horses increased, a significant increase in the pH of the horse meat samples treated with papain was observed. Extending the marinating time from 24 to 48 h for meat samples treated with the papain solution also resulted in a significant increase in the pH of the tested raw meat, to the highest level of 5.64 in horse meat from older animals. An increase in pH after 48 h of marinating was also noted in the other experimental groups, with the exception of the horse meat samples treated with actinidin (no statistically confirmed relationship). A similar relationship was demonstrated for meat samples from young horse carcasses. Extending the marinating time resulted in an increase in the pH value of the horse meat, except for the samples treated with actinidin and bromelain (no statistically confirmed relationships). Istrati et al. [42] demonstrated that meat pH increased with increasing maturation time in both control samples and samples treated with exogenous proteolytic enzymes through injection or marination. The cited authors obtained the highest pH value in beef samples injected with 10% brine with papain added at a concentration of 2 mg/100 g of meat and marinated for 48 h. Plant proteases influence the pH of meat indirectly by breaking down muscle and structural proteins into smaller peptides and free amino acids, some of which are acidic (e.g., glutamic acid, aspartic acid) or alkaline (e.g., lysine, arginine). Therefore, proteolysis products may cause a slight increase or decrease in pH, depending on their chemical nature. The final effect depends on the type of proteins being degraded, the activity of specific proteases, the meat maturation time, and the storage conditions (temperature, oxygen, bacteria, pH) [43]. It should be noted that meat acidity is a key factor influencing oxidative stability. In the present study, the enzyme preparations differed in their initial pH values, with papain, bromelain, actinidin, and the meat tenderizer exhibiting pH values of 6.91, 5.88, 5.42, and 3.29, respectively. However, after marination, the pH values of the horse meat remained within a relatively narrow range (5.46–5.64), suggesting that the buffering capacity of muscle tissue substantially reduced the direct impact of marinade pH. Therefore, although differences in marinade acidity may have contributed to variations in oxidative stability and water-holding properties, the observed effects are likely to be primarily associated with the proteolytic activity and specific characteristics of the individual treatments rather than with the initial pH of the solutions alone. An acidic environment can facilitate the release of pro-oxidant iron from heme proteins and enhance lipid peroxidation reactions, which can consequently lead to increased formation of secondary oxidation products such as TBARSs [26,44].

The pH of meat is crucial for its hydration properties (WHC), i.e., its ability to absorb and retain water within the muscle tissue structure. Considering the data in Table 2, marinating horse meat with plant proteases and culinary tenderizer resulted in a statistically significant increase in thermal drip (*p* < 0.05) compared to the control sample in both age groups. Extending the marinating time of the horse meat samples to 48 h also resulted in a significant increase in the analyzed parameter (*p* < 0.05) in both age groups. The lowest thermal drip was observed for CS after 24 h of marinating in both age groups. Determining the amount of thermal drip is crucial because it provides information about muscle juice losses that may occur as a result of meat heat treatment. The results obtained in this study demonstrate that the use of proteolytic enzymes to marinate horse meat samples resulted in a significant reduction in forced drip (*p* < 0.05) compared to the control sample and samples treated with a culinary tenderizer. The lowest value of the analyzed parameter (*p* < 0.05) was observed for horse meat samples treated with papain, both after 24 and 48 h of marinating in both age groups. As with thermal drip, extending the marinating time led to a significant increase in forced drip in the younger group of horses and in the case of CS and papain-marinated horse meat samples from older animals. Considering the age of the horses, it should be noted that the hydration properties of horse meat are significantly dependent on the age of the horses. The meat obtained from older animals was characterized by significantly lower thermal and forced drip, regardless of the substance used or the marinating time. Considering the effect of plant enzymes on the ability of muscle tissue to absorb and retain water, it should be noted that the available scientific literature does not demonstrate a clear trend. Some researchers report a significant increase in WHC following enzymatic treatment of buffalo [45], duck [46], beef [47], and turkey [48]. In the study by Aminlari et al. [21], actinidin significantly (*p* < 0.05) increased the WHC of beef; the highest increase in WHC (approximately 8%) was observed after incubation of beef with 0.9 units of enzyme/g of beef. Gokoglu et al. [43] also found a relative increase in WHC in squid meat following enzymatic treatment, although this increase was not statistically significant. On the other hand, there are studies demonstrating the opposite trend. In the study by Ketnawa and Rawdkuen [49], when pieces of beef, chicken and squid of uniform size were marinated with bromelain at different concentrations (0%, 3%, 7%, 10% and 20%) for 1 h at room temperature, a decrease in the water retention capacity of the tested samples was observed. The obtained results can be justified by the fact that increasing the dosage and longer treatment of meat with the enzyme preparation results in deeper proteolysis of protein macromolecules along with an increase in the amount of low-molecular-weight hydrolysis products. This reduces hydration and the water content associated with adsorption in meat, impairing its functional and technological properties [50]. The enzyme bromelain hydrolyzes proteins by binding to specific peptide bonds, and its cysteine residue acts as a nucleophile to cleave these bonds. The hydrolysis process breaks down large proteins, primarily collagen and elastin in meat, into smaller peptides and amino acids, thus enabling bromelain to continuously act on other protein molecules [51]. On the other hand, biochemical changes occurring during the hydrolysis process in protein molecules cause the release and cleavage of actomyosin into actin and myosin, which have a better ability to absorb water and become soluble. This may result in an improvement in the WHC of meat. Previous studies conducted on horse meat by Orynbekov et al. [37] show that injecting horse meat with a bromelain solution increased the meat’s ability to retain water. The WHC values of the enzyme-treated meat were statistically significantly higher (*p* < 0.05) compared to the control sample. Injecting the bromelain solution at various concentrations also significantly increased the WHC values (*p* < 0.05) compared to the control sample. Sprinkling horse meat with bromelain powder (2% of the meat weight) also led to a significant increase (*p* < 0.05) in WHC compared to the control sample. In addition, after the use of bromelain powder at amounts of 1%, 3%, 4% and 5%, the WHC values were similar to the WHC values of the control sample of horse meat (*p* > 0.05).

**Figure 1:**
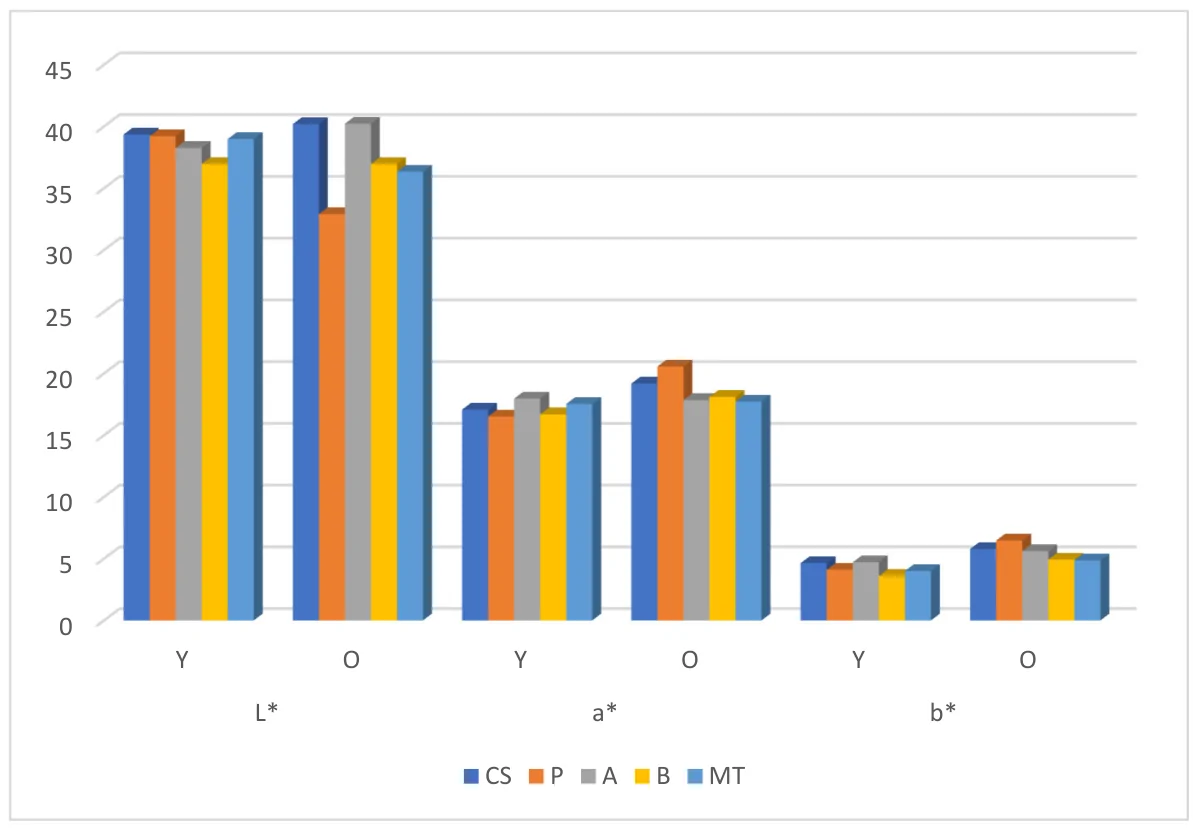
Color parameters of horse meat after 24 h of marinating. O—older horses; Y—younger horses. Statistical significance of differences between means based on ANOVA and *p* values are provided in the tables included in the Supplementary Materials (Table S1). Supplementary materials are contained in Table S4 (Supplementary Materials).

**Figure 2:**
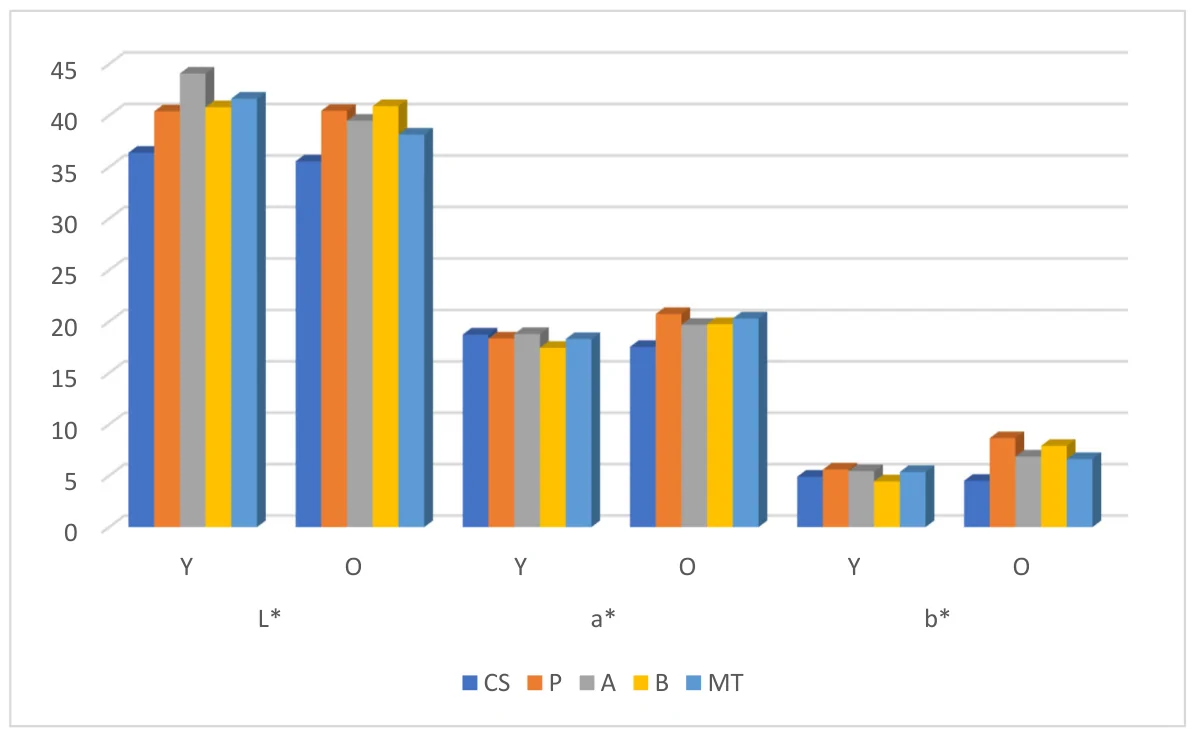
Color parameters of horse meat after 48 h of marinating. O—older horses; Y—younger horses. Statistical significance of differences between means based on ANOVA and *p* values are provided in the tables included in the Supplementary Materials (Table S1). Supplementary materials are contained in Table S4 (Supplementary Materials).

Figure 1 and Figure 2 present data on the color lightness and color parameters of horse meat by the age of the animals, the type of marinating agent used, and the duration of the marinating process. These data indicate that the color lightness of horse meat was statistically significantly influenced by the type of agent used, the interaction effect between age and the type of agent used, and the interaction effect between the type of agent used and the duration of the marinating process (*p* < 0.05). Extending the marinating time of horse meat with plant proteases and culinary tenderizer to 48 h led to an increase in the L* component for all meat samples in both age groups, except for samples from older animals marinated with actinidin. The opposite trend was observed for CS, where the process of storing the meat samples under refrigerated conditions resulted in a significant decrease in the L* component (*p* < 0.05). The resulting increase in the L* value can be explained by the fact that the substances used, through protein degradation, led to a relaxation of the muscle structure, resulting in greater light reflection and increased brightness. The degradation of structural proteins by proteolytic enzymes likely increases the release of myoglobin, which may result in a more intense red color. The redder color of the horse meat samples may be associated with the lower oxidative status observed in the marinated treatments. One possible explanation is a slower conversion of myoglobin to metmyoglobin; this mechanism was not directly investigated in the present study but is supported by the results. After 48 h of marinating the horse meat samples with plant proteases and culinary tenderizer, an increase in the value of the red color component a* was observed (this relationship was not statistically significant). A similar trend was observed for the yellow color value b*. The increase in the b* color parameter could also be caused by slight oxidation or a change in the optical structure of the meat.

It should be noted that the highest values of the a* and b* color components were found in horse meat samples obtained from older animals marinated with papain, both after 24 and 48 h of marinating. Considering the age of the horses, significantly higher values of the a* and b* components were also found in horse meat samples marinated with papain, both after 24 and 48 h of marinating. Furthermore, significantly higher meat color parameters a* and b* (*p* < 0.05) were also found in horse meat samples obtained from older animals marinated for 48 h with bromelain solution. Significantly higher values of the a* color parameter were also found in CS from older animals after 24 h of storage and in samples marinated for 48 h with a culinary tenderizer solution. The increase in the L* component and the a* color parameter of horse meat obtained in this study can be perceived as a positive phenomenon from the consumer’s perspective. The unsatisfactory quality of horse meat is related to the color of the raw meat, defined as dark red with a slight brown tint, which is related to the high content of the muscle pigment myoglobin [52,53]. Previous studies do not provide a clear answer as to how plant proteases shape the color brightness and color parameters of meat. In the study conducted by Abdel-Naeem and Mohamed [54], the L*, a*, and b* values during the formulation of camel burger samples treated with 0.01% papain extract did not differ significantly (*p* > 0.05) from the values of the control samples. Nadzirah et al. [55] showed that the L* and b* values of bromelain-treated beef were significantly higher than those of the control sample, and the a* color component value was lower. Botinestean et al. [56] reported lower L* and b* values for beef steaks treated with papain, bromelain and a mixture of these substances (1:1), but no significant change in a* values was observed. 

Thiobarbituric acid reactive substances (TBARSs) are commonly used as markers of oxidative deterioration, particularly for evaluating the degree of lipid peroxidation in meat systems. In this study, the TBARS values of horse meat were significantly influenced by the marinating agent, marination time, the interaction between animal age and marinating agent, and the three-way interaction among age, marinating agent, and marination time (*p* < 0.05). The results presented in Table 2 show that samples from older animals generally had higher TBARS values, suggesting a greater susceptibility of this type of raw meat to lipid oxidation, most likely due to its higher fat content. The age-related differences in oxidative stability observed in this study can be explained by the combined effect of higher intramuscular fat content and elevated concentrations of heme pigments in older horses. Consequently, meat from older horses is subjected to a dual oxidative stress resulting from both a greater quantity of oxidation-prone lipids and the enhanced catalytic activity of iron-containing compounds. Such conditions promote the initiation of lipid oxidation, which may in turn contribute to the accelerated deterioration of product quality during storage [26,57]. The analysis of the numerical data for the TBARS index showed that the highest values (*p* < 0.05) were shown in the case of CS after 24 and 48 h of marinating in both age groups compared to all other samples of marinated horse meat. Extending the marinating time from 24 to 48 h resulted in a significant increase in the TBARS index (*p* < 0.05) in the CS group, while the opposite trend was observed in all other experimental groups. Changes in the fat oxidation index for the marinated horse meat samples throughout the marinating period showed a steady downward trend in both age groups. The papain-treated samples exhibited the lowest TBARS values in both age groups after both marination periods. During the final marinating period, the TBARS content in these horse meat samples was almost twice as low as that of the control sample. However, antioxidant activity was not directly measured in the present study; therefore, the underlying mechanisms remain hypothetical and are discussed on the basis of previous literature [14,15]. Since the chemical composition of the commercial enzyme preparations was not determined in this study, the observed decrease in TBARS and ORP values cannot be attributed to specific phytochemicals or other non-enzymatic components of the preparations [26]. Therefore, the present study does not allow for the determination of the mechanism responsible for the observed reductions in TBARS and ORP values. Therefore, the mechanisms responsible for the improved oxidative stability observed in protease-treated samples remain to be elucidated in future studies. The relative contribution of each of these mechanisms likely depends on the type of enzyme, the process conditions, and the degree of protein hydrolysis achieved during storage [58,59,60,61,62]. Studies conducted by Elgendy et al. [63] showed that TBARS values gradually increased in chilled ground meat samples treated with papain (50 mg/mL) and bromelain (50 mg/mL), but at a slower rate than in the control group. Significant differences in TBARS values were found between the control group and the groups treated with the specified enzymes.

Table 2 presents data on the oxidation-reduction potential (ORP) of horse meat. High, positive ORP values in meat indicate excess oxidative stress, i.e., an imbalance between oxidants and antioxidants, which leads to unfavorable changes in the meat, such as nutrient degradation and accelerated spoilage. Low, negative ORP values suggest the presence of strong antioxidants and a good oxidation-reduction balance, which is desirable in fresh meat, while positive values indicate oxidation processes and poor meat quality [64]. As shown in Table 2, ORP values in horse meat were significantly affected by the interaction between animal age and marination time, the interaction between age and the type of marinating substance, and the three-way interaction among age, marinating substance, and marination duration. In general, meat from younger horse carcasses was characterized by lower ORP values. These results indicate that a lower redox potential is accompanied by reduced formation of lipid oxidation products, expressed as lower TBARS values, confirming the relationship observed in this study. The age-related differences in oxidative stability observed in this study can be explained by the combined effect of higher intramuscular fat content and elevated concentrations of heme pigments in older horses. Consequently, meat from older horses is subjected to a dual oxidative stress resulting from both a greater quantity of oxidation-prone lipids and the enhanced catalytic activity of iron-containing compounds. Such conditions promote the initiation of lipid oxidation and may, in turn, contribute to the accelerated deterioration of horse meat quality during storage [26,27,28]. The redox potential (ORP) values of horse meat samples with the addition of compounds used in the marinating process were lower compared to the control sample in both age groups. Importantly, the ORP values of marinated horse meat samples in both age groups decreased with marinating time. Significantly lower ORP values (*p* < 0.05) were observed after 48 h of marinating. These results may indicate improved oxidative stability in marinated samples compared with control samples. However, because direct antioxidant activity was not assessed, the specific mechanisms responsible for the decrease in ORP remain unclear.

Samples treated with plant proteases exhibited lower ORP and TBARS values than control samples. This effect may have technological significance, as slowing down oxidative processes helps preserve product quality, including its sensory characteristics and nutritional value. However, it cannot be directly concluded that the observed reduction in TBARS values translates into a specific extension of shelf life. Confirming such a relationship would require a comprehensive storage study involving analysis of multiple time points and the application of defined physicochemical, microbiological, and sensory acceptability criteria. Consequently, the results should be interpreted as indicating improved oxidative stability and the potential for a slower rate of quality deterioration, rather than as direct evidence of extended shelf life.

Correlation analysis revealed a significant positive relationship between ORP and TBARS values in all experimental groups, regardless of animal age, enzyme treatment, or marination time (Table 3). Pearson’s correlation coefficients ranged from 0.453 to 0.798, and all observed associations were statistically significant (*p* < 0.05). These results suggest that higher oxidation–reduction potential was positively associated with the progression of lipid oxidation, as indicated by increased TBARS values.

The observed positive correlations indicate that ORP and TBARS values changed in a consistent manner across the experimental treatments. Because both parameters are associated with oxidative processes, a positive relationship between ORP and TBARS values was expected. 

Notably, stronger correlations were observed after 48 h of marination compared to after 24 h in both young and old horse meat samples. In the younger animals, the correlation coefficient increased from 0.453–0.657 after 24 h to 0.653–0.798 after 48 h, whereas in older horses it increased from 0.468–0.649 to 0.668–0.785.

Correlation coefficients were positive in all treatment groups, indicating a consistent association between ORP and TBARS values regardless of enzyme treatment. These findings indicate that ORP and TBARS values remained positively associated across all treatments and marination periods. 

Interestingly, similar correlation patterns were found in meat from young and old horses, suggesting that animal age had only a limited influence on the fundamental relationship between redox status and lipid oxidation. Although age-related differences in antioxidant capacity and myoglobin content may affect the absolute values of oxidative parameters, the consistent positive correlations indicate that the mechanisms governing oxidation remain comparable across age groups.

Overall, the results indicate a positive association between ORP and TBARS values in horse meat subjected to enzymatic marination.

### 2.3. Texture Parameters

As presented in Table 4, the texture parameters of horse meat were significantly influenced by animal age, the marinating agent applied, the interaction between marinating agent and treatment duration, and the interaction between age and marination time (*p* < 0.05). These factors affected shear force, hardness 1 and 2, stiffness 5 and 8, and chewiness. In general, samples from older horses showed higher shear force values than those obtained from younger animals, indicating greater resistance to cutting. The highest and statistically significant shear force values were recorded for the control samples (CS) and for meat treated with actinidin and bromelain, irrespective of whether marination lasted 24 or 48 h. The trend of increasing shear force values in horse meat with age is confirmed by the scientific literature [65]. As animals age, the protein structure of muscle and connective tissue changes. As animals age, the mechanical stability of connective tissue increases as a result of collagen cross-linking. The compounds used in this study to marinate horse meat resulted in a reduction in shear force compared to the control sample in both age groups, after both 24 and 48 h of marinating. Significantly the lowest shear force values were observed in meat samples treated with papain (*p* < 0.05) after 48 h of marinating. These results are consistent with those reported by other authors. Decreases in shear force values were observed in beef [6], buffalo [45], and duck [46] samples treated with bromelain and papain. In the study by Ionescu et al. [19], the most appropriate treatment conditions involved the application of papain or bromelain at a dose of 10 mg/100 g of meat for 24 h at 4 °C. Such conditions were selected to achieve effective tenderization while limiting excessive structural damage associated with enzymatic proteolysis. Numerous studies have also demonstrated the softening effect of bromelain alone on the muscle tissue of various types of meat. Bromelain extract (BE) alone has been shown to significantly reduce the shear force required to cut beef [49,55,66], poultry [49], and squid [49,67,68] samples compared to control samples. The obtained results can be justified by the fact that enzymatic hydrolysis of meat proteins increases the solubility of free amino groups and hydroxyproline, which may result in a loss of muscle integrity and a reduction in the shear force required to cut the muscle sample or an increase in tenderness [43]. Saengsuk et al. [69] found that restructured pork steak treated with bromelain had 64–80% lower shear force values compared to samples not treated with bromelain. The cited authors attribute these changes to the degradation of myofibrillar and collagen structures. Orynbekov et al. [37] demonstrated that bromelain treatment can effectively reduce the shear force required to cut horse meat samples. The injection of a 5% bromelain solution produced the greatest tenderizing effect, decreasing the shear force to 118 N after 8 h of treatment, compared with the initial value of 270 N, which corresponded to a 56% reduction (*p* < 0.05). A study by Aminlari et al. [21] also demonstrated the usefulness of actinidin in increasing the tenderness of beef. The cited authors noted a 10% decrease in the force required to cut the meat sample when beef slices were exposed to 0.9 actinidin units/g of beef at 37 °C for 2 h.

Three-factor analysis of variance showed that animal age, type of substance used, and marinating time did not significantly affect the adhesiveness, cohesion, elasticity, or resilience of horse meat (Table 4). However, animal age, type of substance used, the interaction effect between type of substance and marinating time, and the interaction effect between age and marinating time significantly influenced hardness 1 and 2, stiffness 8, and chewiness of horse meat (*p* < 0.05). Instrumental analysis of texture parameters showed that the use of a papain solution for marinating horse meat samples resulted in a significant decrease in hardness 1 and 2, stiffness 5 and 8, and chewiness of horse meat (*p* < 0.05) compared to CS and the other experimental groups, in both age groups and after 24 and 48 h of marinating. For example, the hardness values 1 decreased by nearly 45.64 and 42% after 24 h of marinating in both the younger and older horse groups, while the decrease in the analyzed texture parameter after 48 h of marinating with the papain solution was 63 and 60%, respectively. The use of other compounds to marinate the horse meat also resulted in improved texture parameters in both age groups, after both 24 and 48 h of marinating, compared to CS. The most unfavorable texture parameters in both age groups were observed in the control samples of horse meat after 24 h of refrigerated storage. Based on the obtained results, it can be concluded that the use of plant enzymes used in this study and a culinary tenderizer on horse meat contributes to the improvement of the specified texture parameters of the tested raw meat. Reducing the hardness of horse meat may be crucial for consumption of this meat by older people, who often have dental problems and associated difficulties consuming this type of meat. Consuming horse meat tenderized with the substances used in this study may contribute to reducing the protein deficit in this population group. Treating horse meat with enzyme solutions and a culinary tenderizer to tenderize it while promoting its palatability may change the mindset of many meat consumers regarding consuming this type of meat. This information can help meat processors develop strategies to optimize texture-modified horse products in their own businesses. Changes in selected texture parameters indicate the potential to modify the meat’s properties that are crucial for sensory quality and processing. The observed changes in mechanical properties may also have implications for industrial processing. Alterations to hardness, cutting force, or texture profile parameters can affect the raw meat’s suitability for specific unit operations (such as slicing, portioning, comminution, mixing, or forming). However, laboratory results alone do not allow for definitive conclusions regarding reduced equipment load, lower energy consumption, or increased process efficiency. Confirming such benefits requires further research under pilot-scale or industrial conditions, encompassing, among other factors, an assessment of processing efficiency, yield, processing losses, process repeatability, and final product quality.

The improvement in textural parameters due to plant proteases probably stems from their ability to degrade muscle and connective tissue proteins, thereby enhancing product tenderness. Proteases can probably hydrolyze peptide bonds in muscle proteins such as actin and myosin. This leads to a weakening of the muscle fiber structure, increasing the tenderness and softness of the meat. Plant proteases can also break down connective tissue proteins, primarily collagen, so the loosening of the connective tissue makes the meat juicier and easier to chew. Protein hydrolysis improves their ability to bind water, which translates into improved juiciness and consistency of the meat after heat treatment. Kim and Joo [38] also attempted to demonstrate improved texture parameters of horse meat under the influence of plant enzymes. The cited authors exposed horse meat to enzymes, i.e., papain, bromelain, pepsin, and pancreatin, for 1 to 8 h. The obtained results demonstrated that all enzyme solutions improved the hardness and elasticity of the horse meat compared to the control sample. As the exposure time increased from 1 to 8 h, the specified texture parameters of the horse meat were characterized by successively lower values.

### 2.4. Sensory Properties

**Figure 3:**
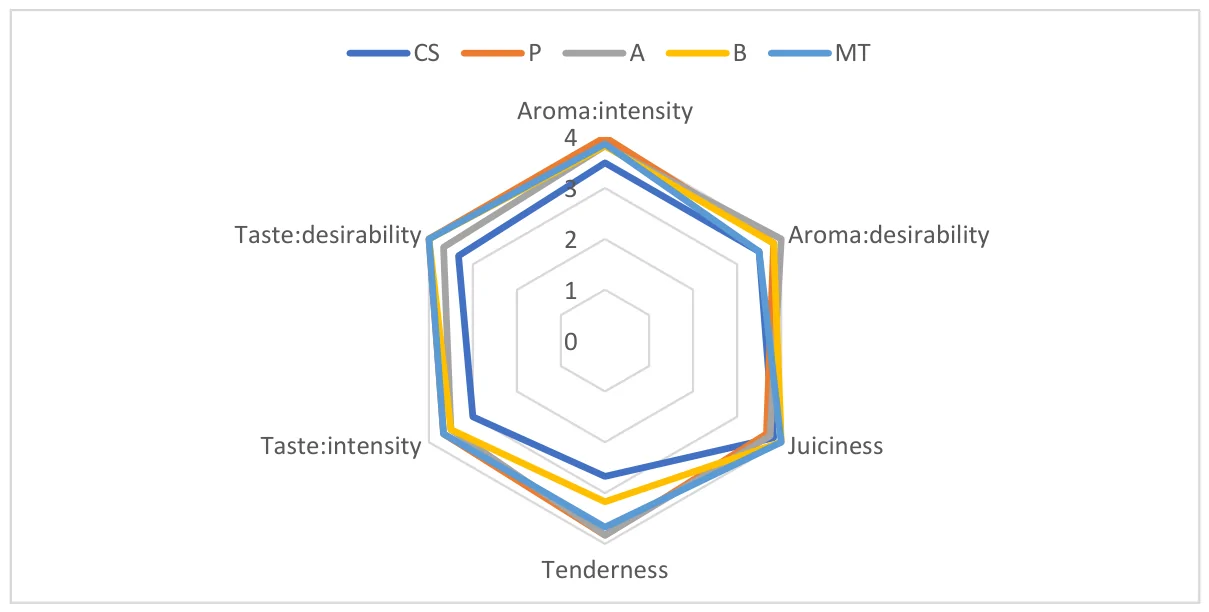
Sensory characteristics of younger horse meat after 24 h of marinating. Statistical significance of differences between means based on analysis of variance and *p* values are presented in the tables included in the Supplementary Materials (Table S2). Supplementary materials are contained in Table S7 (Supplementary Materials).

**Figure 4:**
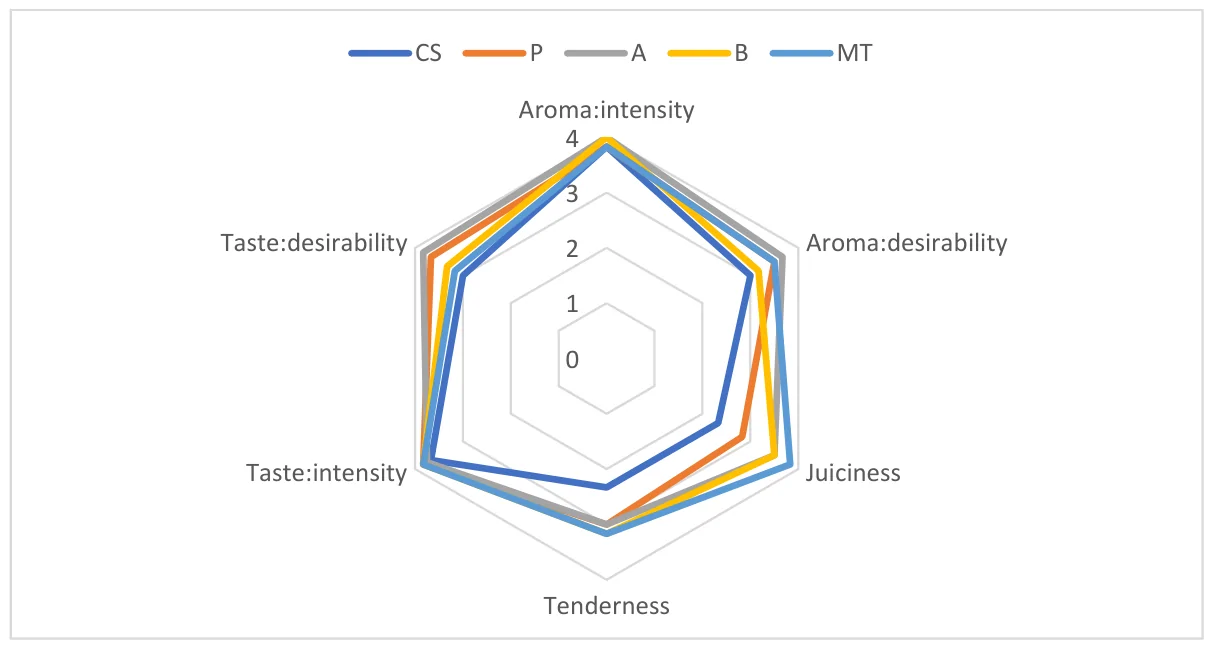
Sensory characteristics of older horse meat after 24 h of marinating. Statistical significance of differences between means based on analysis of variance and *p* values are presented in the tables included in the Supplementary Materials (Table S2). Supplementary materials are contained in Table S7 (Supplementary Materials).

**Figure 5:**
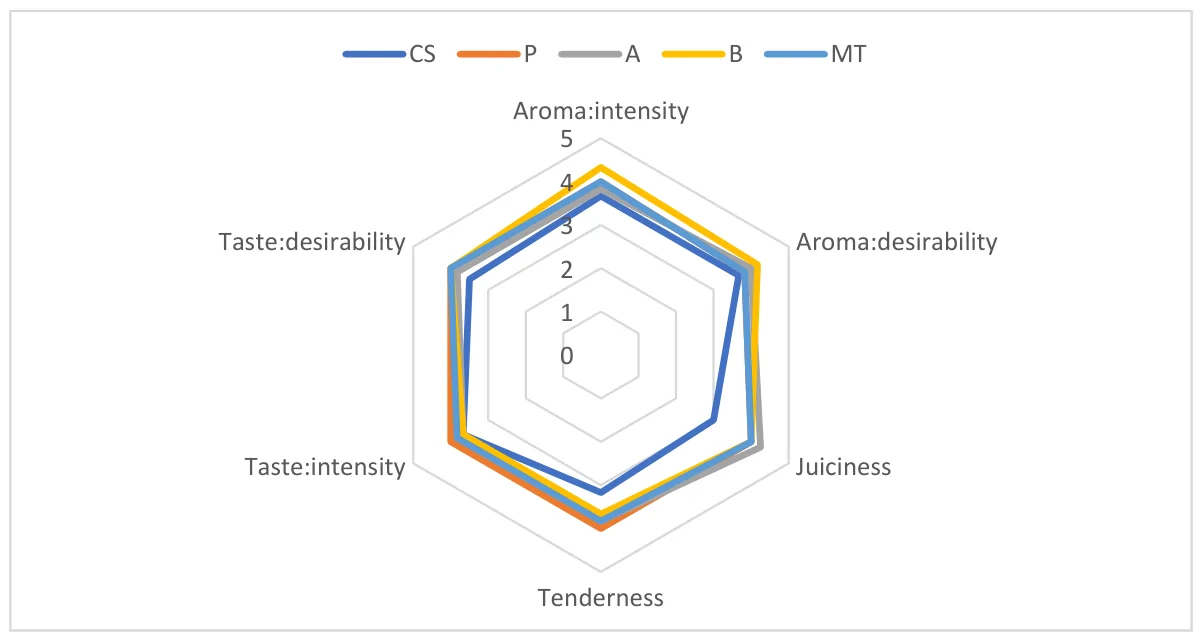
Sensory characteristics of younger horse meat after 48 h of marinating. Statistical significance of differences between means based on analysis of variance and *p* values are presented in the tables included in the Supplementary Materials (Table S2). Supplementary materials are contained in Table S7 (Supplementary Materials).

**Figure 6:**
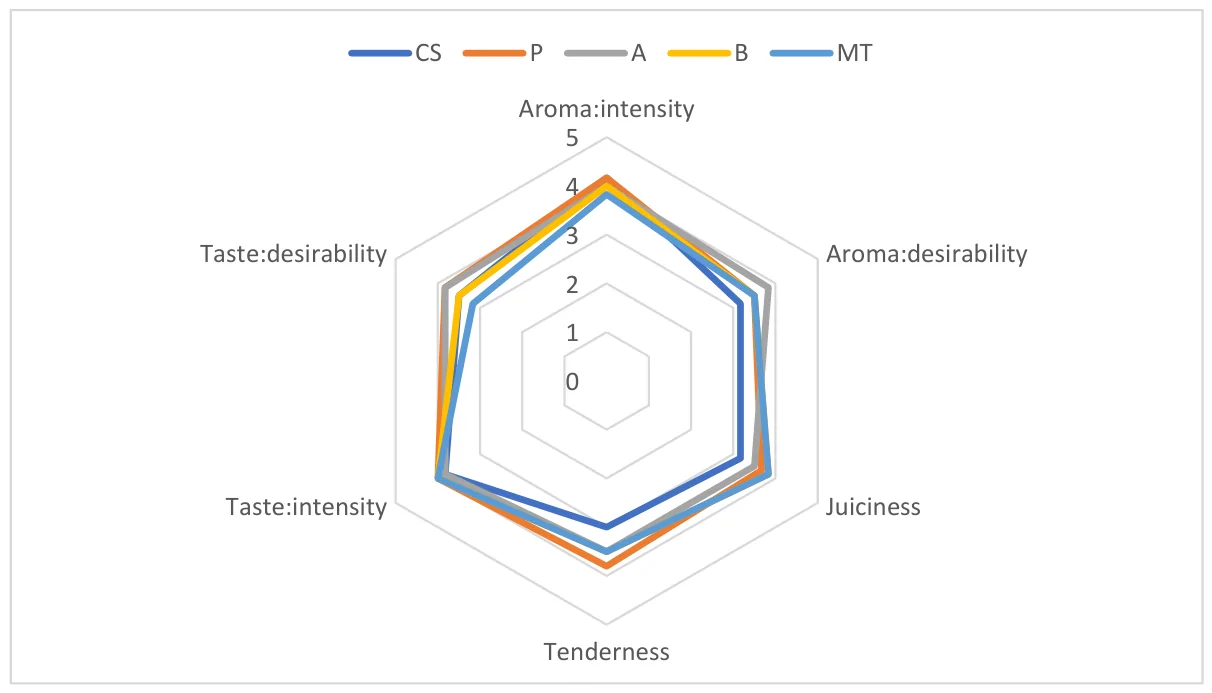
Sensory characteristics of older horse meat after 48 h of marinating. Statistical significance of differences between means based on analysis of variance and *p* values are presented in the tables included in the Supplementary Materials (Table S2). Supplementary materials are contained in Table S7 (Supplementary Materials).

Figure 3, Figure 4, Figure 5 and Figure 6 present data on the sensory quality parameters of the horse meat. These data indicate that the interaction between the type of marinating agent and marinating time, as well as the interaction between age and the type of agent used, had a significant impact on the majority of sensory quality parameters of the horse meat, including aroma (intensity and desirability), juiciness, and tenderness. In addition, the interaction effect between age and the type of substance used significantly influenced the taste of the horse meat, and its intensity and desirability. Furthermore, the age of the animals significantly influenced tenderness, and the desirability based on smell and taste. The type of substance used also significantly influenced taste desirability. 

Regarding the age of the horses, it should be noted that in most experimental groups, in terms of smell and taste desirability, juiciness, and tenderness of horse meat, meat from younger animals achieved higher scores. The opposite trend was observed for taste and smell intensity, where meat samples obtained from older animals received higher scores. Regarding the type of substance used to marinate horse meat, it should be noted that only in the cases of samples treated with the papain or actinidin solution for 24 h of marinating and CS samples after 48 h of storage, meat obtained from younger horse carcasses had lower scores for juiciness. The use of plant proteases and culinary tenderizer resulted in higher scores for all sensory quality parameters compared to CS. Extending the marinating time of horse meat samples to 48 h also resulted in an improvement in most of the analyzed sensory quality parameters of the horse meat. It should be noted that high scores were awarded to horse meat samples after 48 h of marinating for juiciness and taste desirability in the case of samples obtained from younger animal carcasses, as well as for aroma intensity in both age groups. High scores were also awarded for flavor intensity in samples obtained from older animal carcasses. It is likely that the improved sensory properties of the meat after the use of plant proteases are primarily due to changes in the structure, consistency, and chemical composition of the meat, which directly impact consumer perception of taste, smell, and texture. Plant enzymes probably break down muscle fibers and collagen, making the meat more tender and delicate, which is highly appreciated by consumers. This can consequently reduce the sensation of “stringiness” and “toughness” when chewing the meat, making it less rubbery and thereby improving the eating experience.

The improved sensory properties observed after protease treatment may be associated with structural changes within muscle proteins that occur during marination. According to previous studies, proteolysis can contribute to changes in tenderness, juiciness, and flavor development. Therefore, the intensity and attractiveness of the meat’s flavor and aroma may increase. Furthermore, changes in the meat’s structure allow marinades to better penetrate the meat, which may result in a more uniform flavor and intensified sensory experience. The use of marinating compounds may have reduced unpleasant sensory characteristics. In the case of horse meat, improving sensory properties is important because this meat is characterized by a high level of glycogen, which is responsible for the characteristic sweet flavor of this meat [70], which is often considered a less favorable property of horse meat. Some researchers have shown that the use of the enzymes bromelain and papain improved the sensory characteristics of squid [71], buffalo meat [45], and beef [6,72]. The sensory test results of papain-treated beef from Istrati [73] were higher than those of the control meat. The results obtained by Orynbekov et al. [37] indicate that the application of bromelain can positively affect the sensory quality of horse meat. Enzyme-treated samples were evaluated more favorably in terms of tenderness, flavor, and overall palatability compared with the control group. The highest sensory scores were recorded after injection with a 3% bromelain solution, which achieved scores of 5.0 for both texture and aroma. This response was probably related to better penetration and more homogeneous distribution of bromelain throughout the meat tissue, resulting in improved tenderization and flavor formation.

**Figure 7:**
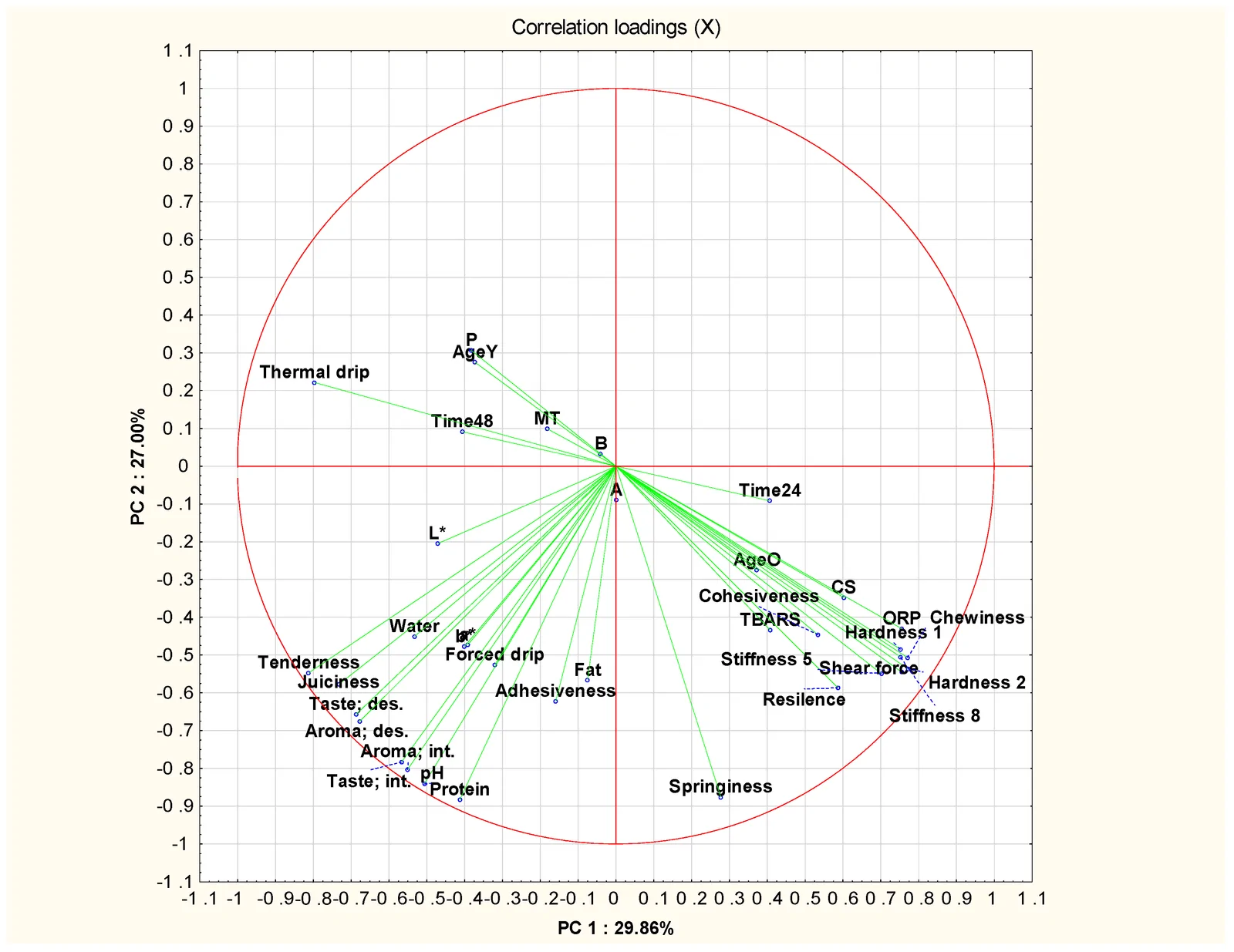
PCA score and correlation loadings (chemical composition, color parameters, physicochemical properties, texture parameters, sensory properties) for horse meat treated with plant proteases.

The PCA results and correlation loadings for the chemical composition, color parameters, texture parameters, physicochemical properties, and sensory properties of horse meat treated with plant proteases are presented in Figure 7.

The PCA visualization based on the first two principal components (which together explain 56.86% of the total variance) enabled a clear characterization of the relationships between technological factors (animal age, marination time, and type of marinating substance) and the quality attributes of horse meat. A clear polarization of samples was observed along PC1 (explains 29.86% of the variance) and PC2 (explains 27.00% of the variance). Instrumentally determined texture attributes, including hardness, stiffness, chewiness, gumminess, resilience, and shear force, together with TBARS and ORP values, were clustered on the right side of the plot. These variables were strongly associated with meat obtained from older horses (AgeO) and with the shorter marination period (Time24). In contrast, sensory quality attributes were located in the lower-left quadrant of the plot, indicating the opposite relationship pattern. Parameters such as tenderness, juiciness, and the desirability and intensity of aroma and taste were closely correlated with higher water and protein contents, and higher pH. Analysis of the qualitative factors also showed that extending the marinating time to 48 h (Time48) and using papain (P) and meat tenderizer (MT) shifted the meat profile toward higher mass losses during heat treatment (thermal drip), which was particularly characteristic of the meat obtained from young horses (AgeY). Due to their proximity to the center of the coordinate system, substances A and B were characterized by the most stable and moderate effect on the rheological and sensory properties of the finished product. 

In summary, the optimization of horse meat marination requires a differentiated technological approach that takes into account both animal age and the desired final product characteristics. For meat obtained from older horses (AgeO), which is naturally characterized by greater hardness, stiffness, chewiness, shear force, and a higher susceptibility to oxidative processes, the application of an extended marination period of 48 h (Time48) appears justified to promote effective loosening of muscle structures and improve tenderness. In contrast, a different strategy should be adopted for meat derived from younger horses (AgeY), where the main technological challenge is the high thermal drip observed during heat treatment. In the case of meat obtained from younger horses (AgeY), reducing the marination period to 24 h (Time24) and limiting the use of papain (P) and meat tenderizer (MT) treatments, which enhanced cooking losses through increased exudate release, may be advantageous. In contrast, actinidin (A) and bromelain (B) treatments appeared to be the most versatile options, as their relatively neutral effects helped maintain the desired sensory profile without causing substantial changes in the rheological properties of the final product. Furthermore, a high initial water and protein content associated with a higher pH value emerged as an important characteristic of the meat, providing favorable conditions for maintaining the juiciness and tenderness of the final culinary product.

## 3. Materials and Methods

### 3.1. Experimental Design

The experimental material comprised samples of the longissimus thoracis muscle collected from 24 horse half-carcasses originating from small-scale farms located in southeastern Poland. The animals were assigned to two age categories: 12 younger horses (2–5 years) and 12 older horses (8–12 years). Age classification was based on information provided in the purchase records. The horses used in the study represented the Małopolski and Silesian breeds, with an average pre-slaughter body weight of 530 ± 30 kg (range: 500–560 kg). Animal age was identified as the primary biological factor, as it is widely considered a key determinant of horse meat quality. Consequently, the animals were grouped by age rather than breed, given that the study’s overarching objective was to evaluate the effects of animal age, plant proteases, a commercial meat tenderizer, and marinating time on the quality characteristics of horse meat. The experimental population consisted of an equal proportion of mares and geldings. The investigators had no involvement with live animals during any stage of the experiment. Following transportation to the slaughter facility, the horses were housed individually for approximately 24 h under standard holding conditions. Prior to slaughter, animals were rendered unconscious using captive-bolt stunning. Slaughter procedures were conducted in accordance with routine industrial practices and complied with current European animal welfare regulations [74]. To evaluate the influence of plant-derived proteases, a commercial meat tenderizer, marination duration, and animal age on horse meat quality, samples weighing approximately 3000 g were excised from the longissimus thoracis muscle between the 13th and 16th thoracic vertebrae. Muscle tissues obtained from both age groups were subsequently aged under refrigerated conditions at 4.0 ± 0.5 °C for 10 days before further experimental processing and analysis.

### 3.2. Meat Processing with Enzymes

Each collected meat sample intended for analysis was cleaned of external fat, connective tissue and tendons, and then divided into 10 batches of steak samples, each with an approximate weight of 300 ± 30 g (12 half-carcasses × 2 marinating periods × 2 age groups × 5 types of treatments = 240 meat samples). The first separated batch of horse meat samples constituted the batch of control samples (CS), which was not subjected to any processing, and the remaining batches of samples were subjected to:

- Papain (P) from ECHT VITAL, which was manufactured in Germany (Leonberg, Germany) and distributed by Sunvita Sp. z o.o. This enzyme was obtained from unripe papaya fruit. The product was a capsule containing 400 mg of 100% powdered papaya extract. The pH of the enzyme combined with water was 6.91. The capsule shell was made of hydroxypropyl methylcellulose. After removing the capsule from the enzyme extract, a solution of 25 mg/100 g of meat was prepared. Pieces of meat to be marinated were placed in glass containers filled with the solution in a 2:1 enzyme:meat ratio.
- Actinidin (A) manufactured by JAR Flavor House (Warsaw, Poland). The pH of the enzyme was 3.46, while the pH of the enzyme combined with water was 5.42. The concentration of the solution was 25 mg/100 g of meat. Pieces of meat intended for marinating were placed in glass vessels filled with the solution in a 2:1 enzyme:meat ratio.
- Bromelain (B) from a pineapple extract purchased from NANGA (Blękwit, Poland), which originated from India. This enzyme had the following characteristics: proteolytic activity of 2500 GDU/g, white powder, and pH in water of 5.88. A solution with a concentration of 25 mg/100 g of meat was prepared. Pieces of meat to be marinated were placed in glass containers filled with the solution at a 2:1 enzyme:meat ratio.
- ASP Culinary Tenderizer (MT) manufactured by ASPOL-SMAK Sp. z o.o. (Rzeszów, Poland). This preparation is available in liquid form and is intended for use in meat products. It contains water, sugar, vinegar, soy protein hydrolysate, lemon juice, flavorings, maltodextrin, and salt. The pH of the preparation was 2.81, and in water, it was 3.29. A 0.5% solution of the preparation was used in the study (500 mg of the commercial product/100 g of meat). Pieces of meat intended for marinating were placed in glass vessels filled with the prepared solution in a 2:1 tenderizer:meat ratio.

The meat samples prepared in this way, together with the control samples, were subjected to laboratory tests after 24 and 48 h of marinating and stored under refrigerated conditions (temperature: 4 °C ± 0.5 °C).

The biological experimental unit was the individual horse half-carcass. The study included 24 half-carcasses belonging to two age groups (12 younger horses and 12 older horses. Samples obtained from each half-carcass were divided among all treatment variants and marination periods. Therefore, each treatment combination was represented by independent biological observations originating from individual animals within each age group. Thus, for each treatment variant, observations were obtained from 12 independent biological units (animals) within each age group. One sample (3000 g) was collected from each half-carcass and divided into 10 subsamples (5 for 24 h marinating time and 5 for 48 h marinating time. All laboratory determinations were performed in triplicate; however, the analytical replicates were used solely to improve measurement precision and were averaged prior to statistical analysis. Consequently, analytical replicates were not considered independent experimental observations.

### 3.3. Analytical Methods

The determination of water content was performed following the requirements of PN-ISO 1442:2000 [75]. Protein content was analyzed using the Kjeldahl procedure, whereby the nitrogen concentration obtained during the assay was converted into total protein in accordance with PN-75/A-04018 [76]. The lipid fraction of the samples was determined by Soxhlet extraction, following the PN-ISO 1444:2000 standard [77]. Meat pH was recorded using an OSH 12–01 electrode coupled with a CPC-411 pH meter produced by ELMETRON, Zabrze, Poland. The measurements were taken with an accuracy of 0.01 pH units after calibration of the device with buffer solutions of pH 4.00 and 7.00. Color attributes of the meat were expressed as L*, a*, and b* coordinates in the CIE LAB color space. The analysis was conducted using the reflectance technique with an NR20XE device (3nh Technology Co., Ltd., Shenzhen, China), following the methodological approach reported in [23]. Lipid peroxidation was evaluated using the thiobarbituric acid reactive substance (TBARS) assay according to the procedure described by Pikul et al. [78]. This method estimates the accumulation of secondary lipid oxidation products, particularly malondialdehyde (MDA), through their reaction with thiobarbituric acid. During incubation under elevated temperature conditions, aldehydic compounds generated from lipid degradation form chromogenic adducts with the reagent. The absorbance of the resulting complexes was subsequently measured at 532 nm using a Spekol 2000 spectrophotometer (Analytik Jena AG, Germany). Quantitative assessment was performed relative to a control reference sample. The redox status of the meat samples was assessed by measuring the oxidation–reduction potential (ORP) following the methodology reported by Stanisławczyk et al. [79]. An ERPt-13 redox electrode connected to an ELMETRON CPC-505 multifunction meter (ELMETRON, Zabrze, Poland) was employed for all determinations. Readings were taken after the electrode response reached equilibrium. To express the results as Eh values referenced to the standard hydrogen electrode, the correction factor corresponding to the reference electrode potential (211 mV at 20 °C) was applied. For ORP analysis, 10 g of previously ground meat sample was combined with 50 mL of distilled water and homogenized using an ULTRA-TURRAX T25 system (IKA, Germany) equipped with an S25N-18G dispersing element at 15.000 rpm for 1 min. The homogenization procedure was performed at 20 °C on material passed through a 3 mm grinding plate. The redox potential of the prepared homogenate was subsequently recorded at 20 °C with an analytical precision of 0.01 mV. The hydration properties of the meat were characterized through measurements of thermal and forced drip losses. Thermal drip was determined following the methodology of Janicki and Walczak [80], which provides information on water losses occurring during heat treatment. In addition, forced drip was evaluated according to the Grau and Hamm procedure [81], which reflects the water-holding capacity of muscle tissue under applied pressure. Instrumental determination of raw meat tenderness was performed by measuring the shear force using a TA.XTplus Texture Analyzer (Stable Micro Systems Ltd., Surrey, UK). Cylindrical specimens (1 cm diameter) were excised parallel to the muscle fiber orientation using a cork borer. The samples were subsequently sheared with a Warner–Bratzler attachment equipped with a V-shaped blade. The maximum force required to cut through the muscle tissue was recorded and expressed as N/cm^2^. Instrumental texture characteristics of the meat samples were evaluated by means of Texture Profile Analysis (TPA). Measurements were carried out using a CT3-25 Texture Analyzer (Brookfield Engineering Laboratories, WI, USA) equipped with a cylindrical probe measuring 38.1 mm in diameter and 20 mm in length. Cubic samples (20 × 20 × 20 mm) were prepared from each experimental batch and analyzed at 4 °C. Samples were subjected to a double-compression test to 50% of their original height. The test speed and return speed were set at 2 mm/s, the trigger load was 0.80 N, and the interval between compression cycles was 2 s. Texture parameters, including hardness 1, hardness 2, stiffness at 5 mm, stiffness at 8 mm, adhesiveness, resilience, cohesiveness, springiness, gumminess, and chewiness, were automatically calculated using Texture Pro CT software (V.1.9 Build 39; Brookfield, WI, USA). All texture determinations were performed according to the procedure reported by Stanisławczyk et al. [82]. The sensory properties of horse meat samples were evaluated as described by Baryłko-Pikielna and Matuszewska [83]. Briefly, 100 g portions of each sample were steam-cooked at 95 °C until an internal temperature of 80 ± 2 °C was reached, which was monitored using a digital needle-probe thermometer (Sous Vide Thermapen; MERA, Warsaw, Poland). After cooking, the samples were cooled to 20 ± 2 °C and cut perpendicular to the muscle fibers into slices approximately 1.5 cm thick. The samples were placed in disposable plastic containers with lids, coded using random three-digit numbers, and presented to the sensory panel in a randomized order. The sensory characteristics of horse meat were assessed by a trained panel consisting of 12 assessors selected according to the requirements of ISO 8586:2023 [84]. Evaluations were carried out in a sensory laboratory designed in compliance with ISO 8589 [85], following the general principles specified in ISO 8587:2006 [86]. Sample quality was scored using a five-point hedonic scale covering aroma intensity, aroma acceptability, flavor intensity, flavor acceptability, juiciness, and tenderness, as previously described by Stanisławczyk et al. [82]. Detailed definitions of each rating level for aroma intensity, aroma desirability, flavor intensity, flavor desirability, juiciness, and tenderness are presented in Table 5. 

All assessors had prior experience in the sensory evaluation of meat and meat products. Assessments were performed in individual sensory booths under standardized white lighting and controlled environmental conditions. The laboratory was maintained at a room temperature of 20 ± 2 °C and was free from distracting noise and extraneous odors. Evaluations were conducted during daytime hours to ensure consistent assessment conditions throughout the study. Test samples were presented in a randomized order to minimize potential bias. The sensory panel was composed of equal numbers of male and female participants, with ages ranging from 38 to 55 years. To prevent carry-over effects between samples, panelists rinsed their mouths with mineral water and observed a 30 s interval before proceeding to the next evaluation. Sensory evaluation was conducted in ten independent sessions, each including 24 samples under identical environmental and serving conditions to improve assessment consistency.

### 3.4. Statistical Analysis

The statistical evaluation was based on 24 independent biological replicates (horse half-carcasses) divided between two age groups. Samples from each biological replicate were assigned to five treatment variants and two marination periods, resulting in a total of 240 experimental samples. All analytical determinations were performed in triplicate, and the triplicate values were averaged before statistical analysis. The collected data were subjected to statistical processing after grouping according to the experimental factors. Data analysis was carried out using Statistica 13.3PL software (TIBCO Software Inc., Palo Alto, CA, USA). The distribution of the data was assessed for normality using the Kolmogorov–Smirnov test, while the homogeneity of variances was evaluated with the Brown–Forsythe test. Selected physicochemical properties, chemical composition, texture parameters, and color and sensory evaluations of meat were analyzed by three-way analysis of variance (ANOVA) using the GLM procedure in Statistica (STATISTICA version 13.3; Stat Soft, Kraków, Poland), which treated the substance used, age or marinating time as a fixed effect and the carcass half as a random term. Whenever significant differences were detected, mean values were analyzed using Tukey’s post hoc honestly significant difference (HSD) test at a significance level of *p* < 0.05. Tukey’s method is more conservative than the LSD test, but less so than Scheffé’s test. Individual comparisons are rejected less frequently when using Tukey’s method than with the LSD method. Thus, this test is the most recommended test for comparing pairs of means (as it allows for the formation of homogeneous groups). The Type I error rate here is lower than that of other tests (LSD, Duncan’s, Newman–Keuls). The results are presented as means accompanied by their corresponding standard errors (Table 1, Table 2 and Table 4). To explore the association between oxidation–reduction potential (ORP) and lipid oxidation intensity, expressed as TBARS values, Pearson’s correlation coefficients were calculated. Correlation analyses were performed independently for each age group, marination period, and treatment condition. Statistical significance was accepted at *p* < 0.05 (Table 3). Principal component analysis (PCA) was performed to reduce dimensionality and identify latent data structures. To conduct a multidimensional analysis of the relationships between the studied parameters, a complete data matrix was created, encompassing variables related to chemical composition, color parameters, physicochemical properties, texture parameters, and sensory evaluation results. Each test object constituted an observation (row), while each measurement characteristic constituted a separate variable (column). Before analysis, a complete data preparation procedure was performed, including checking for missing data, identifying outliers, and assessing variable distributions. When significant distribution asymmetry was observed, appropriate variance-stabilizing transformations were applied. Due to the heterogeneous nature of the variables and their different measurement scales, all quantitative variables were standardized using the z-score method according to Equation (1):

(1) $$z_{i}=\frac{x_{i}-\bar{x}}{s}$$

where z_i_ is the normalized value (transformed to a dimensionless scale); x_i_ is the value of the variable for the ith observation; $\bar{x}$ is the arithmetic mean of the variable calculated from all samples; and s is the standard deviation of the variable calculated from all samples, which ensures equal participation of each variable in the PCA model. Analysis was performed on the correlation matrix, in accordance with recommendations for data with diverse units and value ranges. Qualitative variables, such as age, time, and type of substance, were coded as binary variables (0/1) according to the rules of binary coding, allowing for their inclusion in the multivariate analysis without introducing collinearity. The number of principal components was selected based on eigenvalues, the scree plot, and the total percentage of explained variance. Interpretation of the results was based on the analysis of factor loadings, which determined the contribution of individual variables to the formation of the components, and item scores, which represented the distribution of samples in the factor space. The relationships between variables and their impact on the data structure are presented in a two-dimensional PCA plot that includes the first two principal components. The direction and length of the variable vectors were interpreted as a measure of the strength and nature of the correlation with a given component. Variables with similar directions and close proximity to each other indicated strong positive correlations, while variables with opposite directions indicated negative correlations. The plot considered the dominant spectral bands of individual variables, enabling precise identification of the parameters that most differentiated the analyzed samples (Figure 7).

## 4. Conclusions

The results of this study provide valuable information regarding the potential application of plant proteases (papain, bromelain, and actinidin) and a commercial meat tenderizer in the meat industry. Horse age significantly affects meat quality—meat from older animals typically contains more fat, less water, and is tougher and more susceptible to lipid oxidation. Plant proteases improve horse meat quality, particularly in terms of tenderness, texture, and color. Papain has the strongest effect, as it softens the meat most, especially after 48 h of marinating. Extending marinating to 48 h increases the effectiveness of enzymes, improving tenderness and color intensity. Meat from older horses responds more strongly to enzymes, significantly improving its texture. The hydration properties of the meat change because enzymes increase thermal drip but reduce forced drip, thus improving water binding in the raw state. Sensory evaluation confirmed the effectiveness of enzymes, particularly papain and bromelain, which increase tenderness, juiciness, and palatability. Meat from older horses requires longer marinating (48 h) and responds best to papain. However, meat from young horses should be marinated for a shorter period (24 h) to minimize thermal bleeding. Actinidin and bromelain are the most stable and predictable enzymes, as they do not cause excessive texture changes or bleeding.

## Figures and Tables

**Table 1:** The chemical composition of horse meat by the age of the horse, the type of substance used and the length of the marinating time (mean ± SE).

Specification	Age	Marinating Time: 24 h					Marinating Time: 48 h					ANOVA	*p*-Value
		CS	P	A	B	MT	CS	P	A	B	MT		
Fat (%)	Y	2.53 ^x^ ± 0.15	2.97 ^x^ ± 0.89	2.90 ^x^ ± 0.81	2.73 ^x^ ± 0.32	3.10 ^x^ ± 0.10	2.80 ^x^ ± 0.10	2.77 ^x^ ± 0.11	2.77 ^x^ ± 0.05	2.87 ^x^ ± 0.30	2.53 ^x^ ± 0.15	A	0.0001
	O	3.73 ^y^ ± 0.05	3.57 ^y^ ± 0.25	4.40 ^y^ ± 0.69	3.93 ^y^ ± 1.13	3.90 ^y^ ± 0.10	3.57 ^y^ ± 0.15	4.33 ^y^ ± 0.49	3.20 ^y^ ± 0.17	3.53 ^y^ ± 0.15	3.70 ^y^ ± 0.10		
Water (%)	Y	75.37 ^x^ ± 0.15	75.03 ^x^ ± 0.73	74.97 ^x^ ± 0.66	75.23 ^x^ ± 0.20	74.93 ^x^ ± 0.11	75.17 ^x^ ± 0.05	75.17 ^x^ ± 0.05	75.27 ^x^ ± 0.05	75.13 ^x^ ± 0.30	75.40 ^x^ ± 0.10	A	0.0014
	O	74.20 ^y^ ± 0.01	74.13 ^y^ ± 0.41	73.83 ^y^ ± 0.55	74.23 ^y^ ± 1.00	74.05 ^y^ ± 1.00	74.47 ^y^ ± 0.15	73.90 ^y^ ± 0.43	74.60 ^y^ ± 0.17	74.53 ^y^ ± 0.11	74.37 ^y^ ± 0.11		
Protein (%)	Y	20.70 ± 0.05	20.50 ± 0.23	20.30 ± 0.37	20.50 ± 0.05	20.60 ± 0.05	20.43 ± 0.01	20.33 ± 0.01	20.50 ± 0.01	20.50 ± 0.05	20.50 ± 0.05		
	O	20.73 ± 0.01	20.67 ± 0.10	20.97 ± 0.10	20.67 ± 0.58	20.63 ± 0.10	20.70 ± 0.05	20.70 ± 0.11	20.70 ± 0.01	20.67 ± 0.01	20.73 ± 0.10		

Notes: ^xy^ different letters in the same column indicate statistically significant differences between the age groups for a particular period of storage (*p* < 0.05); ANOVA: three-way ANOVA. A—age of animal; Y—younger horses, O—older horses. S, substance; T, time; CS—control samples; P—papain; A—actinidin; B—bromelain; MT—meat tenderizer. Supplementary materials for ANOVA are contained in Table S3 (Supplementary Materials).

**Table 2:** Physicochemical properties of horse meat by the age of the horse, the type of substance used and the length of the marinating time (mean ± SE).

Specification	Age	Marinating Time: 24 h					Marinating Time: 48 h					ANO-VA	*p*-Value
		CS	P	A	B	MT	CS	P	A	B	MT		
pH	Y	5.48 ^ab^ ± 0.01	5.46 ^ax^ ± 0.01	5.49 ^ab^ ± 0.01	5.50 ^ab^ ± 0.01	5.46 ^a^ ± 0.01	5.54 ^b^ ± 0.04	5.52 ^abx^ ± 0.01	5.48 ^ab^ ± 0.02	5.47 ^ab^ ± 0.01	5.51 ^ab^ ± 0.01	A × S × T	0.0001
	O	5.49 ^ac^ ± 0.01	5.55 ^ay^ ± 0.03	5.52 ^ac^ ± 0.02	5.46 ^c^ ± 0.05	5.49 ^ac^ ± 0.01	5.55 ^a^ ± 0.01	5.64 ^by^ ± 0.05	5.51 ^ac^ ± 0.02	5.52 ^ac^ ± 0.01	5.54 ^ac^ ± 0.03		
TBARSs [mg MDA/kg]	Y	1.00 ^ax^ ± 0.01	0.75 ^bx^ ± 0.01	0.83 ^c^ ± 0.01	0.85 ^dx^ ± 0.01	0.90 ^e^ ± 0.01	1.12 ^fx^ ± 0.01	0.58 ^jx^ ± 0.01	0.60 ^hx^ ± 0.01	0.61 ^i^ ± 0.01	0.58 ^j^ ± 0.01	T; S; A × T; A × S × T	0.0001
	O	1.23 ^ay^ ± 0.01	0.84 ^by^ ± 0.01	0.88 ^ca^ ± 0.01	0.95 ^dy^ ± 0.01	0.92 ^e^ ± 0.01	1.40 ^fy^ ± 0.01	0.75 ^hy^ ± 0.01	0.76 ^hy^ ± 0.01	0.64 ^i^ ± 0.01	0.62 ^j^ ± 0.01		
ORP [mV]	Y	306.00 ^ax^ ± 0.01	288.00 ^b^ ± 0.01	292.00 ^b^ ± 0.01	300.00 ^ax^ ± 0.01	302.00 ^a^ ± 0.01	309.00 ^ax^ ± 0.01	242.00 ^cx^ ± 0.01	272.00 ^d^ ± 0.01	274.00 ^d^ ± 0.01	242.00 ^cx^ ± 0.01	S × T; A × S; A × S × T	0.0001
	O	328.00 ^ay^ ± 0.01	297.00 ^b^ ± 0.01	301.00 ^b^ ± 0.01	323.00 ^ay^ ± 0.01	306.00 ^b^ ± 0.01	335.00 ^ay^ ± 0.01	287.00 ^by^ ± 0.01	289.00 ^b^ ± 0.01	286.00 ^b^ ± 0.01	276.00 ^by^ ± 0.01		
Forced drip [cm^2^]	Y	5.90 ^ax^ ± 0.26	4.01 ^bx^ ± 0.25	5.65 ^cx^ ± 0.22	5.60 ^cx^ ± 0.48	6.60 ^dx^ ± 0.46	7.63 ^ex^ ± 0.36	5.37 ^fx^ ± 0.25	6.37 ^gx^ ± 0.15	6.60 ^dx^ ± 0.30	8.73 ^hx^ ± 0.05	S; A; S × T; A × T; A × S	0.0001
	O	5.70 ^ay^ ± 0.31	2.97 ^by^ ± 0.46	5.00 ^cy^ ± 0.65	5.10 ^cy^ ± 0.01	6.17 ^dy^ ± 0.66	6.60 ^ey^ ± 0.55	4.77 ^fy^ ± 0.40	5.23 ^cy^ ± 0.51	5.13 ^cy^ ± 0.36	6.26 ^dy^ ± 0.45		
Thermal drip [%]	Y	37.64 ^ax^ ± 0.10	40.25 ^bx^ ± 0.39	41.75 ^bx^ ± 0.86	40.69 ^bx^ ± 0.76	40.79 ^bx^ ± 0.34	41.44 ^bx^ ± 0.02	45.83 ^cx^ ± 0.61	45.39 ^cx^ ± 0.76	46.46 ^cx^ ± 0.60	45.18 ^cx^ ± 0.62	S; A; T; A × S × T	0.0011
	O	29.99 ^ay^ ± 0.59	35.25 ^by^ ± 0.44	36.54 ^by^ ± 0.24	35.58 ^by^ ± 0.67	36.99 ^by^ ± 0.61	37.75 ^by^ ± 0.84	40.50 ^cy^ ± 0.88	41.60 ^cy^ ± 0.05	40.09 ^cy^ ± 0.17	42.65 ^cy^ ± 0.55		

Notes: ^abcdefghij^ values indicated by different letters in the rows show statistically significant differences across the different types of marinating substance used (*p* < 0.05); ^xy^ different letters in the same column indicate statistically significant differences between the age groups for a particular period of storage (*p* < 0.05); ANOVA: three-way ANOVA. A—age of animal; Y—younger horses, O—older horses; S—substance; T—time; CS—control samples; P—papain, A—actinidin, B—bromelain, MT—meat tenderizer; ORP—oxidation–reduction potential. Supplementary materials for ANOVA are contained in Table S5 (Supplementary Materials).

**Table 3:** Pearson’s correlation between ORP and TBARS values in horse meat samples.

Specification	Age	Marinating Time: 24 h					Marinating Time: 48 h				
		CS	P	A	B	MT	CS	P	A	B	MT
Pearson’s correlation; r	Y	0.453	0.657	0.633	0.621	0.611	0.653	0.798	0.754	0.731	0.722
	O	0.468	0.649	0.648	0.634	0.629	0.668	0.785	0.741	0.711	0.708
*p* Value	Y	0.048	0.011	0.030	0.035	0.027	0.046	0.019	0.039	0.035	0.023
	O	0.037	0.023	0.037	0.029	0.034	0.039	0.025	0.042	0.037	0.031
Confidence interval	O	0.0388–0.7344	0.0467–0.9101	0.0056–0.9028	0.2701–0.8263	0.2551–0.8211	0.0397–0.9089	0.3384–0.9502	0.2368–0.9382	0.1878–0.9318	0.1694–0.9292
	Y	0.0578–0.7430	0.0327–0.9077	0.0310–0.9074	0.0073–0.9031	0.2822–0.8304	0.0662–0.9134	0.3072–0.9467	0.2088–0.9346	0.1473–0.9261	0.1414–0.9252

Notes: A—age of animal; Y—younger horses; O—older horses; CS—control samples; P—papain; A—actinidin; B—bromelain; MT—meat tenderizer; ORP—oxidation–reduction potential.

**Table 4:** Texture parameters of horse meat by the age of the horse, the type of substance used and the length of marinating time (mean ± SE).

Specification	Age	Marinating Time: 24 h						Marinating Time: 48 h				ANO- VA	*p*-Value
		CS	P	A	B	MT	CS	P	A	B	MT		
Shear force [N/cm^2^]	Y	64.74 ^ax^ ± 3.71	48.07 ^b^ ± 4.90	51.99 ^bx^ ± 5.57	49.70 ^bx^ ± 7.07	50.68 ^b^ ± 6.12	62.78 ^ax^ ± 6.29	43.55 ^c^ ± 2.99	50.33 ^bx^ ± 5.73	48.39 ^bx^ ± 4.86	48.70 ^b^ ± 4.95	A; S; S × T; A × T	0.0001
	O	92.21 ^ay^ ± 4.27	52.94 ^b^ ± 9.91	73.90 ^cy^ ± 6.48	66.38 ^dy^ ± 5.40	55.92 ^b^ ± 2.59	81.75 ^ey^ ± 4.42	47.94 ^f^ ± 5.82	67.36 ^dy^ ± 4.86	58.20 ^by^ ± 2.94	53.76 ^b^ ± 3.93		
Hardness 1 [N]	Y	136.59 ^ax^ ± 9.65	74.25 ^dx^ ± 7.08	105.39 ^bx^ ± 9.46	103.34 ^bx^ ± 6.52	96.29 ^b^ ± 5.29	133.73 ^ax^ ± 5.67	49.49 ^e^ ± 7.15	99.71 ^bx^ ± 5.04	93.84 ^cx^ ± 5.41	87.21 ^c^ ± 5.63	A; S; S × T; A × T	0.0001
	O	148.82 ^ay^ ± 9.31	86.27 ^by^ ± 6.51	128.55 ^cy^ ± 7.25	114.77 ^dy^ ± 6.58	102.16 ^e^ ± 6.98	140.81 ^gy^ ± 8.37	55.97 ^f^ ± 4.22	117.64 ^dy^ ± 6.75	103.74 ^ey^ ± 5.71	94.71 ^b^ ± 4.53		
Hardness 2 [N]	Y	79.11 ^xa^ ± 6.84	34.73 ^dx^ ± 3.09	57.43 ^bx^ ± 4.54	51.08 ^cx^ ± 4.32	54.40 ^cb^ ± 4.87	64.55 ^bx^ ± 4.40	27.44 ^d^ ± 2.74	49.92 ^ex^ ± 3.65	49.66 ^ex^ ± 4.14	42.64 ^e^ ± 3.24	A; S; S × T; A × T	0.0002
	O	104.68 ^ay^ ± 5.06	42.29 ^dy^ ± 1.46	67.59 ^by^ ± 1.46	59.21 ^by^ ± 1.44	57.13 ^b^ ± 1.99	77.50 ^cy^ ± 2.35	33.66 ^e^ ± 1.15	59.57 ^by^ ± 2.07	59.17 ^by^ ± 2.08	48.34 ^d^ ± 1.85		
Stiffness 5 [N]	Y	24.35 ^a^ ± 2.32	6.45 ^cx^ ± 0.55	15.33 ^b^ ± 1.09	13.51 ^b^ ± 0.84	12.90 ^b^ ± 0.45	19.65 ^d^ ± 0.93	5.68 ^c^ ± 0.30	13.25 ^b^ ± 0.81	12.21 ^b^ ± 0.91	10.43 ^b^ ± 0.81	S; A × S	0.0008
	O	25.35 ^a^ ± 2.41	10.02 ^cy^ ± 1.14	16.26 ^b^ ± 0.52	15.76 ^b^ ± 0.98	13.22 ^b^ ± 0.53	20.85 ^d^ ± 0.70	6.40 ^c^ ± 0.52	13.42 ^b^ ± 0.83	13.15 ^b^ ± 0.86	10.77 ^b^ ± 0.52		
Stiffness 8 [N]	Y	92.79 ^ax^ ± 2.56	24.70 ^bx^ ± 0.60	67.23 ^cx^ ± 0.48	59.55 ^cx^ ± 0.77	57.21 ^c^ ± 1.22	88.40 ^ax^ ± 1.46	15.45 ^d^ ± 0.92	59.90 ^cx^ ± 2.39	53.87 ^cx^ ± 1.05	33.79 ^e^ ± 0.68	A; S; S × T; A × T	0.0001
	O	113.63 ^ay^ ± 2.98	33.12 ^by^ ± 0.58	85.58 ^cy^ ± 1.03	73.49 ^dy^ ± 0.74	58.30 ^e^ ± 1.09	98.74 ^fy^ ± 1.88	18.76 ^g^ ± 0.81	74.29 ^dy^ ± 2.14	64.64 ^ey^ ± 1.94	34.67 ^b^ ± 0.17		
Adhesiveness (mJ)	Y	1.70 ± 0.41	1.20 ± 0.15	1.73 ± 0.36	1.67 ± 0.60	1.27 ± 0.13	1.20 ± 0.66	1.57 ± 0.98	1.33 ± 0.15	1.27 ± 0.02	1.30 ± 0.94		
	O	1.97 ± 0.36	1.30 ± 0.80	1.80 ± 0.15	1.73 ± 0.06	1.60 ± 0.76	1.63 ± 0.35	1.77 ± 0.43	1.57 ± 0.98	1.60 ± 0.65	1.33 ± 0.51		
Cohesive- ness	Y	0.24 ± 0.13	0.17 ± 0.33	0.30 ± 0.02	0.22 ± 0.09	0.18 ± 0.15	0.16 ± 0.03	0.20 ± 0.09	0.19 ± 0.10	0.18 ± 0.15	0.14 ± 0.05		
	O	0.37 ± 0.02	0.18 ± 0.02	0.34 ± 0.07	0.29 ± 0.07	0.25 ± 0.03	0.29 ± 0.05	0.30 ± 0.07	0.22 ± 0.10	0.16 ± 0.05	0.14 ± 0.08		
Springiness [mm]	Y	4.91 ± 0.41	2.43 ± 2.57	4.83 ± 0.54	4.21 ± 0.32	4.10 ± 0.19	4.73 ± 0.11	2.23 ± 0.48	3.58 ± 1.03	3.11 ± 0.89	3.76 ± 0.43		
	O	5.76 ± 0.49	3.39 ± 0.43	5.04 ± 1.04	4.23 ± 0.63	4.44 ± 0.32	5.53 ± 0.73	3.16 ± 2.56	4.21 ± 2.23	3.93 ± 0.62	3.49 ± 2.26		
Resilience	Y	0.19 ± 0.11	0.11 ± 0.10	0.17 ± 0.03	0.14 ± 0.08	0.13 ± 0.06	0.16 ± 0.04	0.15 ± 0.10	0.11 ± 0.06	0.13 ± 0.04	0.11 ± 0.06		
	O	0.25 ± 0.04	0.15 ± 0.01	0.22 ± 0.06	0.20 ± 0.04	0.21 ± 0.02	0.23 ± 0.05	0.20 ± 0.08	0.15 ± 0.08	0.17 ± 0.04	0.16 ± 0.04		
Chewiness [mJ]	Y	147.90 ^ax^ ± 6.94	38.40 ^b^ ± 4.17	93.63 ^cx^ ± 8.66	85.23 ^dx^ ± 6.50	85.37 ^dx^ ± 5.35	127.23 ^e^ ± 5.58	29.20 ^bx^ ± 2.15	76.83 ^fx^ ± 5.66	53.97 ^gx^ ± 4.49	49.90 ^gx^ ± 2.66	A; S; S × T; A × T	0.0001
	O	192.90 ^ay^ ± 9.22	41.20 ^b^ ± 4.07	113.60 ^cy^ ± 8.33	100.73 ^dy^ ± 7.61	110.30 ^dy^ ± 6.04	134.50 ^e^ ± 6.97	37.73 ^by^ ± 2.09	90.63 ^fy^ ± 3.87	99.33 ^fy^ ± 6.08	96.83 ^fy^ ± 4.13		

Notes: ^abcdefg^ values indicated by different letters in a row show statistically significant differences across the different types of marinating substance used (*p* < 0.05); ^xy^ different letters in the same column indicate statistically significant differences between the age groups for a particular period of storage (*p* < 0.05); ANOVA: three-way ANOVA. A—age of animal; Y—younger horses; O—older horses; S—substance; T—time; CS—control samples; P—papain; A—actinidin; B—bromelain; MT—meat tenderizer. Supplementary materials for ANOVA are contained in Table S6 (Supplementary Materials).

**Table 5:** Sensory assessment chart.

Score	Aroma Intensity	Aroma Desirability	Flavor Intensity	Flavor Desirability	Juiciness	Tenderness
1	Very weak or imperceptible	Not desirable	Very weak or imperceptible	Not desirable	Very dry	Very hard
2	Weak	Slightly undesirable	Weak	Slightly undesirable	Slightly dry	Slightly hard
3	Moderate	Neither desirable nor undesirable	Moderate	Neither desirable nor undesirable	Moderately juicy	Moderately tender
4	Strong	Desirable	Strong	Desirable	Juicy	Tender
5	Very strong	Highly desirable	Very strong	Highly desirable	Very juicy	Very tender

## Data Availability

The original contributions presented in the study are included in the article; further inquiries can be directed to the corresponding author.

## Supplementary Materials

The following supporting information can be downloaded at: https://www.mdpi.com/article/10.3390/molecules31183236/s1, Table S1: Color parameters of horse meat depending on the age of the horses, the type of substance used and the length of marinating time (means ± SE); Table S2: Sensory properties of horse meat depending on the age of the horses, the type of substance used and the length of marinating time (means ± SE); Table S3: The chemical composition of horse meat depending on the age of the horses, the type of substance used and the length of marinating time; Table S4: Color parameters of horse meat depending on the age of the horses, the type of substance used and the length of marinating time; Table S5: Physicochemical properties of horse meat depending on the age of the horses, the type of substance used and the length of marinating time; Table S6: Texture parameters of horse meat depending on the age of the horses, the type of substance used and the length of marinating time; Table S7: Sensory properties of horse meat depending on the age of the horses, the type of substance used and the length of marinating time.
