# Supplementary material for: Effect of Plant Proteases on Oxidative Stability, Physicochemical and Sensory Properties of Horse Meat from Different Age Groups During Marination
Source: Molecules. 2026 Sep 13;31(18):3236. doi: 10.3390/molecules31183236 (PMC13609070; doi:10.3390/molecules31183236)
Supplement: Supplementary file 1 [file molecules-31-03236-s001.zip › molecules-4470549-supplementary.pdf]

Table S1. Color parameters of horse meat depending on the age of the horses, the type of substance used and the length of marinating time (means±SE).

Specification	Age	Marinating time 24 hours					Marinating time 48 hours					ANOVA	*p*-Value
		CS	P	A	B	MT	CS	P	A	B	MT		
L*	Y	39.35^{bc}±0.96	39.22^{bcx}±0.86	38.26^{c}±0.02	36.96^{ac}±0.10	38.98^{cx}±0.70	36.39^{a}±0.40	40.41^{b}±0.91	44.08^{bx}±0.71	40.81^{b}±0.54	41.65^{bx}±0.65	S; A×S; S×T	0.0003
	O	40.19^{b}±0.20	32.90^{ay}±0.40	40.22^{b}±0.74	36.96^{a}±0.37	36.33^{ay}±0.05	35.56^{a}±0.47	40.47^{b}±0.57	39.51^{by}±0.48	40.92^{b}±0.44	38.16^{by}±0.57		
a*	Y	17.07^{x}±0.65	16.51^{x}±0.46	17.96±0.47	16.69±0.60	17.53±0.50	18.74±0.82	18.35^{x}±0.92	18.79±0.54	17.45^{x}±0.26	18.30^{x}±0.11	A×T	0.0007
	O	19.18^{y}±0.39	20.56^{y}±0.72	17.84±0.36	18.10±0.92	17.72^{a}±0.58	17.54±0.17	20.73^{y}±0.19	19.68±0.67	19.75^{y}±0.31	20.30^{by}±0.11		
b*	Y	4.64±0.25	4.10^{x}±0.36	4.71±0.19	3.60±0.41	4.00±0.56	4.90±0.20	5.60^{x}±0.27	5.45±0.49	4.46^{x}±0.42	5.37±0.39	A×T; A×S	0.0003
	O	5.78^{ab}±0.76	6.47^{aby}±0.53	5.61^{ab}±0.27	4.92^{ab}±0.72	4.86^{a}±0.10	4.51^{a}±0.28	8.66^{cy}±0.92	6.87^{ab}±0.26	7.91^{acy}±0.89	6.62^{ab}±0.97		

Explanatory notes: ^{abc} values indicated by different letters in the rows show statistically significant differences across the types of marinating substance used ($p < 0.05$); ^{xy} different letters in the same column indicate statistically significant differences between the age groups of horses in particular periods of storage ($p < 0.05$); ANOVA: three-way ANOVA; A - age of animal; Y - younger horses, O - older horses. S, substances; T, time; CS – control samples; P – papain, A – actinidin, B – bromelain, MT - meat tenderizer

Table S2. Sensory properties of horse meat depending on the age of the horses, the type of substance used and the length of marinating time (means±SE).

Specification	Age	Marinating time 24 hours					Marinating time 48 hours					ANOVA	*p*-Value
		CS	P	A	B	MT	CS	P	A	B	MT		
Aroma: intensity	Y	3.50^{ax}±0.25	4.00^{db}±0.86	3.83^{b}±0.15	3.83^{b}±0.04	3.87^{b}±0.50	3.67^{ax}±0.50	4.00^{db}±0.01	3.83^{b}±0.28	4.33^{cx}±0.57	4.00^{db}±0.28	S×T; A ×S	0.0004
	O	3.83^{ay}±0.76	3.83^{a}±0.57	4.00^{ac}±0.28	4.00^{ac}±0.28	3.83^{a}±0.76	4.17^{cy}±0.28	4.17^{c}±0.76	4.00^{ac}±0.86	4.00^{acy}±0.50	3.83^{a}±0.28		
Aroma: desirability	Y	3.50^{ax}±0.76	3.83^{bx}±0.28	4.00^{dx}±0.20	3.83^{bx}±0.04	3.50^{a}±0.50	3.67^{abcx}±0.50	3.83^{bx}±0.28	4.00^{d}±0.28	4.17^{dx}±0.28	3.83^{bx}±0.28	A; A ×S; S×T	0.0003
	O	3.00^{ay}±1.04	3.50^{by}±0.50	3.67^{bcy}±0.28	3.17^{ay}±0.50	3.50^{b}±0.50	3.17^{ay}±0.86	3.50^{by}±0.15	3.83^{c}±0.20	3.50^{by}±0.28	3.50^{by}±0.04		
Juiciness	Y	3.83^{bcx}±0.04	3.67^{bx}±0.25	3.75^{bc}±0.86	4.00^{cdx}±0.01	4.00^{cd}±0.57	3.00^{a}±0.76	4.00^{cdx}±1.04	4.25^{dx}±0.76	4.00^{cd}±0.28	4.00^{cd}±0.28	A ×S; S×T	0.0001
	O	2.33^{ay}±0.50	2.83^{by}±0.76	3.50^{c}±0.86	3.50^{cy}±0.04	3.83^{d}±0.76	3.17^{e}±0.76	3.67^{cy}±0.57	3.50^{cy}±0.50	3.83^{d}±0.50	3.83^{d}±0.76		
Tenderness	Y	2.67^{ax}±0.15	3.83^{bdx}±0.04	3.83^{bdx}±0.04	3.17^{c}±0.28	3.67^{bx}±0.28	3.17^{c}±0.28	4.00^{d}±0.01	3.83^{bdx}±0.28	3.67^{b}±0.57	3.83^{bdx}±0.15	A; A ×S; S×T	0.0001
	O	2.33^{ay}±0.76	3.00^{by}±0.50	3.00^{by}±0.04	3.17^{b}±0.60	3.17^{by}±0.28	3.00^{b}±0.01	3.80^{c}±0.28	3.50^{dy}±0.76	3.50^{d}±0.57	3.50^{dy}±0.86		
Taste: intensity	Y	3.00^{ax}±0.50	3.67^{bd}±0.57	3.50^{b}±0.50	3.50^{bx}±0.50	3.67^{bd}±0.57	3.67^{bd}±0.28	4.00^{c}±0.01	3.67^{bd}±0.28	3.67^{bdx}±0.57	3.83^{cd}±0.28	A ×S	0.0011
	O	3.67^{ay}±0.28	3.83^{ab}±0.76	3.75^{ab}±0.01	3.83^{aby}±0.76	3.83^{ab}±0.50	3.83^{ab}±0.28	4.00^{b}±0.01	3.83^{a}±0.57	4.00^{by}±0.50	4.00^{b}±0.57		
Taste: desirability	Y	3.33^{ax}±0.57	4.00^{bx}±0.10	3.67^{c}±0.57	4.00^{bx}±0.50	4.00^{bx}±0.01	3.50^{a}±0.50	4.00^{b}±0.01	3.83^{bc}±0.57	4.00^{bx}±0.01	4.00^{bx}±0.50	A; S; A ×S;	0.0001
	O	3.00^{ay}±0.50	3.67^{bdy}±0.57	3.83^{b}±0.57	3.33^{cdy}±0.52	3.17^{ay}±0.28	3.50^{d}±0.86	3.83^{b}±0.15	3.83^{b}±0.50	3.50^{dy}±0.50	3.17^{ay}±0.28		

Explanatory notes: ^{abcde} values indicated by different letters in the rows show statistically significant differences across the types of marinating substance used ($p < 0.05$); ^{xy} different letters in the same column indicate statistically significant differences between the age groups of horses in particular periods of storage ($p < 0.05$); ANOVA: three-way ANOVA; A - age of animal; Y - younger horses, O - older horses. S, substances; T, time; CS – control samples; P – papain, A – actinidin, B – bromelain, MT - meat tenderizer

Table S3. The chemical composition of horse meat depending on the age of the horses, the type of substance used and the length of marinating time.

Specification	ANOVA	*p*-Value	df	F value	Effect sizes
Fat (%)	A	0.0001	1	19.89	0.365
Water (%)	A	0.0014	1	8.95	0.155
Protein (%)	-	-	-	-	-

ANOVA: three-way ANOVA; A—age of animal; S—substance; T—time; - lack of interaction

Table S4. Color parameters of horse meat depending on the age of the horses, the type of substance used and the length of marinating time.

Specification	ANOVA	*p*-Value	df	F value	Effect sizes
L*	S;	0.0003	4	16.25	0.246
	A×S;	0.0003	4	15.97	0.176
	S×T	0.0003	4	16.04	0.205
a*	A×T	0.0007	1	13.55	0.199
b*	A×T;	0.0003	1	15.89	0.690
	A×S	0.0003	4	16.12	0.706

ANOVA: three-way ANOVA; A—age of animal; S—substance; T—time

Table S5. Physicochemical properties of horse meat depending on the age of the horses, the type of substance used and the length of marinating time.

Specification	ANOVA	*p*-Value	df	F value	Effect sizes
pH	A×S×T	0.0001	4	20.02	0.155
TBARS	T;	0.0001	1	19,99	0.656
[mg MDA/kg]	S;	0.0001	4	20.12	0.563
	A×T;	0.0001	1	22.32	0.602
	A×S×T	0.0001	4	21.76	0.587
ORP [mV]	S×T;	0.0001	4	20,04	0,297
	A×S;	0.0001	4	19.89	0.312
	A×S×T	0.0001	4	23.03	0.356
Forced drip	S;	0.0001	4	20.34	0.891
[cm^2]	A;	0.0001	1	19,98	0.765
	S×T;	0.0001	4	22.34	0.768
	A×T;	0.0001	1	23.87	0.832
	A×S	0.0001	4	21.16	0.754
Thermal drip [%]	S;	0.0011	4	9.78	0.312
	A;	0.0011	1	8.67	0.245
	T;	0.0011	1	9.13	0.298
	A×S×T	0.0011	4	9.56	0.345

ANOVA: three-way ANOVA; A—age of animal; S—substance; T—time

Table S6. Texture parameters of horse meat depending on the age of the horses, the type of substance used and the length of marinating time.

Specification	ANOVA	*p*-Value	df	F value	Effect sizes
Shear force [N/cm²]	A;	0.0001	1	20.23	0.432
	S;	0.0001	4	19.79	0.496
	S×T;	0.0001	4	23.06	0.476
	A×T	0.0001	1	22.14	0.487
Hardness 1 [N]	A;	0.0001	1	20.67	0.178
	S;	0.0001	4	22.65	0.566
	S×T;	0.0001	4	20.87	0.478
	A×T	0.0001	1	19.69	0.356
Hardness 2 [N]	A;	0.0002	1	18.65	0.278
	S;	0.0002	4	17.78	0.526
	S×T;	0.0002	4	16.98	0.354
	A×T	0.0002	1	18.23	0.289
Stiffness 5 [N]	S;	0.0008	4	12.12	0.586
	A×S	0.0008	4	11.78	0.487
Stiffness 8 [N]	A;	0.0001	1	20.05	0.289
	S;	0.0001	4	21.45	0.546
	S×T;	0.0001	4	22.98	0.456
	A×T	0.0001	1	20.65	0.356
Adhesiveness [mJ]	-	-	-	-	-
Cohesiveness	-	-	-	-	-
Springiness [mm]	-	-	-	-	-
Resilence	-	-	-	-	-
Chewiness [mJ]	A;	0.0001	1	20.45	0.378
	S;	0.0001	4	21.22	0.526
	S×T;	0.0001	4	19.69	0.456
	A×T	0.0001	1	22.03	0.387

ANOVA: three-way ANOVA; A—age of animal; S—substance; T—time; - lack of interaction

Table S7. Sensory properties of horse meat depending on the age of the horses, the type of substance used and the length of marinating time.

Specification	ANOVA	*p*-Value	df	F value	Effect sizes
Aroma: intensity	S×T;	0.0004	4	14.43	0.173
	A×S	0.0004	4	13.89	0.213
Aroma: desirability	A;	0.0003	1	15.12	0.189
	A×S;	0.0003	4	15.98	0.267
	S×T	0.0003	4	15.67	0.234
Juiciness	A×S;	0.0001	4	20.45	0.463
	S×T	0.0001	4	22.56	0.387
Tenderness	A;	0.0001	1	19.98	0.378
	A×S;	0.0001	4	23.45	0.432
	S×T	0.0001	4	21.23	0.423

Taste: intensity	A×S	0.0011	4	9.23	0.253
Taste: desirability	A;	0.0001	1	9.23	0.169
	S;	0.0001	4	8.63	0.249
	A×S	0.0001	4	8.78	0.189

ANOVA: three-way ANOVA; A—age of animal; S—substance; T—time
